# Accurate determination of genotypic variance of cell wall characteristics of a *Populus trichocarpa* pedigree using high-throughput pyrolysis-molecular beam mass spectrometry

**DOI:** 10.1186/s13068-021-01908-y

**Published:** 2021-03-06

**Authors:** Anne E. Harman-Ware, David Macaya-Sanz, Chanaka Roshan Abeyratne, Crissa Doeppke, Kathleen Haiby, Gerald A. Tuskan, Brian Stanton, Stephen P. DiFazio, Mark F. Davis

**Affiliations:** 1 Renewable Resources and Enabling Sciences Center, National Renewable Energy Laboratory, Golden, CO 80401 USA; 2 Department of Biology, West Virginia University, Morgantown, WV 26506 USA; 3 Greenwood Resources, Portland, OR 97201 USA; 4 Biosciences Division, Oak Ridge National Laboratory, Oak Ridge, TN 37830 USA; 5 Biosciences Center, National Renewable Energy Laboratory, Golden, CO 80401 USA

**Keywords:** Biomass composition, Poplar, Heritability, Pyrolysis-molecular beam mass spectrometry, Phenotypic plasticity

## Abstract

**Background:**

Pyrolysis-molecular beam mass spectrometry (py-MBMS) analysis of a pedigree of *Populus trichocarpa* was performed to study the phenotypic plasticity and heritability of lignin content and lignin monomer composition. Instrumental and microspatial environmental variability were observed in the spectral features and corrected to reveal underlying genetic variance of biomass composition.

**Results:**

Lignin-derived ions (including *m/z* 124, 154, 168, 194, 210 and others) were highly impacted by microspatial environmental variation which demonstrates phenotypic plasticity of lignin composition in *Populus trichocarpa* biomass. Broad-sense heritability of lignin composition after correcting for microspatial and instrumental variation was determined to be *H*^2^ = 0.56 based on py-MBMS ions known to derive from lignin. Heritability of lignin monomeric syringyl/guaiacyl ratio (*S*/*G*) was *H*^2^ = 0.81. Broad-sense heritability was also high (up to *H*^2^ = 0.79) for ions derived from other components of the biomass including phenolics (e.g., salicylates) and C5 sugars (e.g., xylose). Lignin and phenolic ion abundances were primarily driven by maternal effects, and paternal effects were either similar or stronger for the most heritable carbohydrate-derived ions.

**Conclusions:**

We have shown that many biopolymer-derived ions from py-MBMS show substantial phenotypic plasticity in response to microenvironmental variation in plantations. Nevertheless, broad-sense heritability for biomass composition can be quite high after correcting for spatial environmental variation. This work outlines the importance in accounting for instrumental and microspatial environmental variation in biomass composition data for applications in heritability measurements and genomic selection for breeding poplar for renewable fuels and materials.

**Supplementary Information:**

The online version contains supplementary material available at 10.1186/s13068-021-01908-y.

## Background

Biomass cell wall composition plays an important role in the potential use of lignocellulosic feedstocks for renewable fuels and materials. In particular, total lignin content and monomer composition can impact the technical and economic feasibility of using lignocellulosic biomass such as wood as a feedstock for biofuels or other goods [1–3]. Modifying lignin content or composition could be accomplished in several ways, including genetic engineering [4–8] or environmental priming [9, 10]. Lignin content can also be modified through breeding approaches, taking advantage of natural variation to obtain significant trait gains, using well-established protocols and experimental designs [11, 12]. Moreover, recent sophisticated techniques such as genomic selection allow for accelerated breeding with considerable reduction in costs [7, 13, 14].

Trait heritability is the cornerstone for breeding, such that a trait with no heritability is by definition unresponsive to selection, and therefore, not amenable for breeding [15]. On the contrary, traits with high heritability display large selection gains with reduced effort. However, heritability is a not an absolute parameter, since it is codependent on the population and the experiment where it is measured [16, 17]. First, the degree of genetic control over a given trait in the population under study will determine the theoretical upper limit of heritability. Second, traits heavily affected by environmental conditions (phenotypic plasticity) will display lower heritability when measured in trials with poorly controlled environmental variation (microspatial variation) or when estimated across several trials with a range of environmental conditions (geographical variation) [17].

Accurate and affordable measurement of a trait is crucial for breeding in general and for heritability estimation in particular. Although precise methods to estimate lignin content and composition using wet chemistry are available, they are time-consuming and not practical for large sample sets [18]. High- and moderate-throughput methods such as near-infrared spectroscopy (NIR), pyrolysis–gas chromatography (py-GC/MS), and pyrolysis-molecular beam mass spectrometry (py-MBMS) have demonstrated the ability to rapidly measure biomass composition for a variety of studies and applications [9, 19, 20].

In particular, py-MBMS has been used as a high-throughput technique to estimate relative lignin content, lignin monomeric syringyl (S) to guaiacyl (G), (S/G) ratios, sugar composition and terpenoid content of lignocellulosic biomass [9, 10, 21–25]. The source of ions present in mass spectra derived from the pyrolysis of lignocellulosic biomass has been the focus of many investigations and has been studied on many types of instruments and scales [26–29]. Typically, py-MBMS spectra are sum (total ion chromatogram, TIC) or mean-normalized and are used to screen or elucidate cell wall composition on the basis of a small fraction of the ions present in the spectra. For example, lignin content can be estimated by regression or relative to a standard (response factor method using a single standard) by summation of ion intensities, particularly *m/z* 120 (H), 124 (G), 137 (G), 138 (G), 150 (H, G), 152, 154 (S), 164 (G), 167 (S), 168 (S), 178 (G), 180, 181, 182 (S), 194 (S), 208 (S) and 210 (S), where H denotes coumaryl (H) monomer-derived species, G refers to species typically derived from coniferyl monomers and S derives from sinapyl monomers [1]. Relative S/G ratios can be estimated based on the abundance of ions known to originate from corresponding monomers. Large datasets of hundreds of samples have been analyzed in an effort to elucidate the underlying spectral data structure and its relationship to biomass composition using basic data analytics for quality control, dimension reduction and data projection, modeling and prediction. For example, principle component analysis (PCA) or discriminant analysis (DA) can be used to differentiate or group samples on the basis of py-MBMS spectra [30, 31]. Partial least squares (PLS) regression models have been used to predict composition based on data collected from wet chemistry and other spectroscopic methods [22, 24, 32]. One study used features in py-MBMS spectra as phenotypes to identify genes related to lignin biosynthesis in poplar using a novel multi-omic “lines of evidence” approach [33]. Ions and spectral features in the context of cell wall chemistry have also been used as traits in conjunction with genetic mapping approaches and heritability studies of poplar and other hardwoods originating from various genetic backgrounds and growing conditions [1, 3, 34]. However, thorough data analytics approaches to comprehensively analyze py-MBMS spectra from large lignocellulosic sample sets with a detailed analysis of the sources of spectral trends are lacking. As interpretation of py-MBMS spectral data in a biologically meaningful way is not trivial, particularly with respect to genetic and environmental effects, a comprehensive spectral analysis method needs to be employed for proper data interpretation.

Typically, focus has been placed on the relative abundance of a small fraction of py-MBMS ions with many assumptions in place and lack of detail outlining the variation observed in the rest of the spectra and corresponding variance attributed to instrumental drift and from metadata associated with field trials. With proper experimental design, large data sets of py-MBMS spectra within a single species consisting of various genetic or environmentally impacted traits could potentially be efficiently analyzed to incorporate quality control, predictions of composition and properties but to also enable the generation of new hypotheses regarding the structure of the spectra as it relates to biomass properties and phenotypic plasticity.

In this study, we have analyzed the wood cell wall composition of a large full factorial 7 × 7 pedigree of *Populus trichocarpa* with a high level of technical and biological replication (*n* = 2721 ramets). The goal of the study was to partition the variance of the py-MBMS output given the instrumental error, the microspatial environmental heterogeneity throughout a field trial and the genotype and familial identities of the samples. We address both the ion intensities produced by py-MBMS analysis and the compound estimations of lignin content and S/G ratio derived from appropriate ions in the spectra. This work also elucidates common trends among the ions due to microspatial environments and genotypic identity.

## Results

### Assessment of instrumental error and correction of py-MBMS data

Due to the unprecedented size of the of the analysis set, quality control (QC) assessment was needed during analysis, particularly since the condition of the instrument changed between replicate analyses. Analysis of spectra from 6 types of standards monitored individually indicated no particular time-dependent trend, indicating differences were likely based on changes in instrument noise attributed to fluctuations in ion energy and the conditions of the path of pyrolysis vapors (Additional file 1 Table S1; Figs. S1–2). The estimated uncorrected lignin content was also reproducible between measurement replicates (Pearson correlation = 0.87; *R*^2^ = 0.75; Additional file 1 Table S2). The standard deviation of lignin content between replicates ranged from 0.0 to 1.7 wt% lignin, all being less than 10% of the mean determined value. However, since there was minor spectral drift over the replicates of the population that was consistent among standards based on PCA and variance analysis (Additional file 1: Fig. S3), ions were subsequently corrected for “tray effect.” Most ions with high variance attributed to tray effects were not used in calculations for lignin composition or S/G ratio (the exceptions being *m/z* 167 and 181) and otherwise attributed to “noise” as outlined in the “Discussion” section.

### Effect of microspatial environmental variation on py-MBMS spectra

**Fig. 1:**
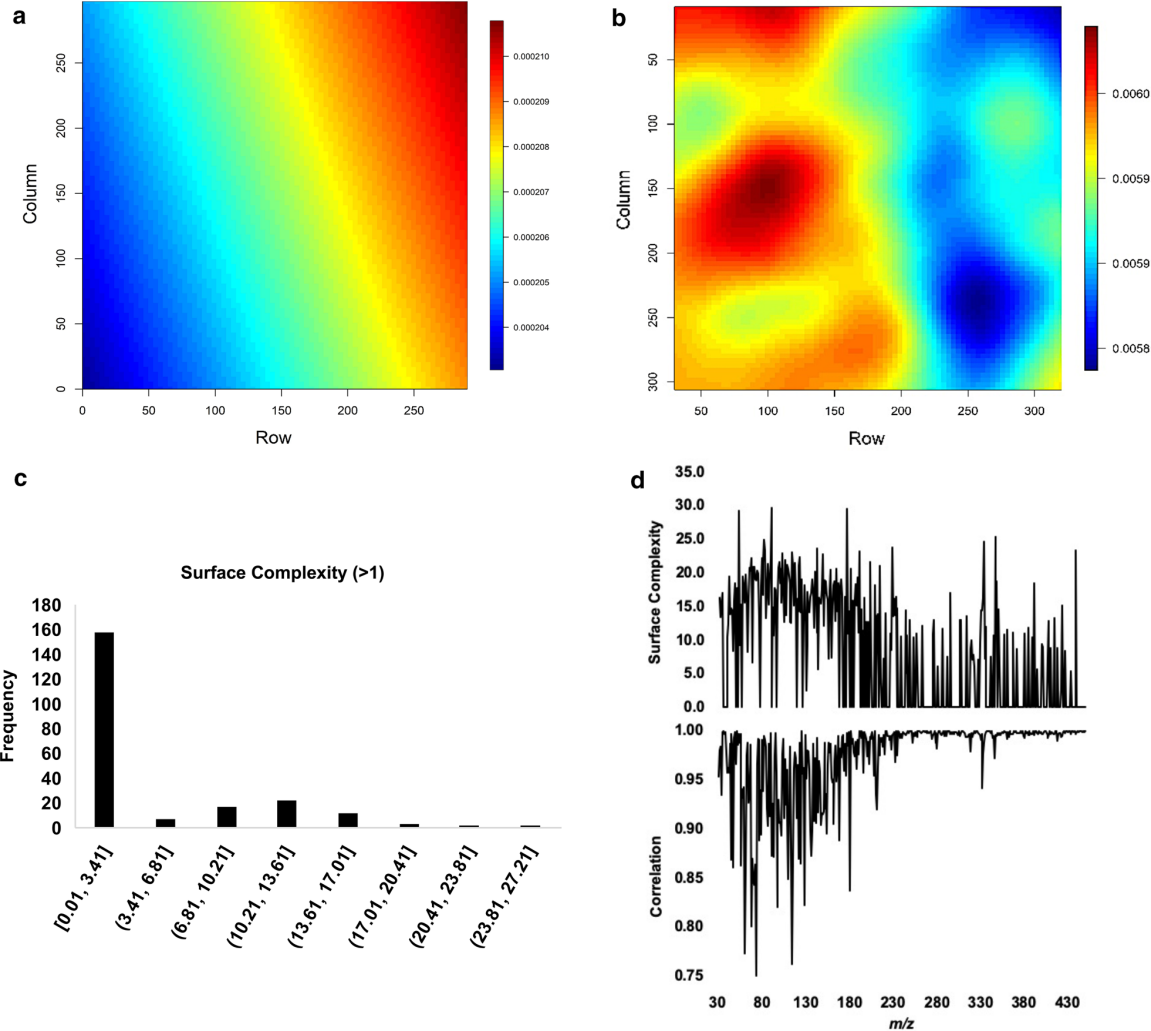
(**a**) Simple surface from thin plate spline (TPS) analysis for null surface complexity (SC) parameters (ion, such as *m/z* 38 shown here, or phenotype not impacted by microspatial environment) where environment corresponds to row position and column position (m) in the field and (**b**) complex surface for SC parameters > 1 indicative of microspatial impacts on the ions (*m/z* 32 shown here) or phenotype, (**c)** histogram showing distribution of SC > 1 for ions in MBMS spectra where [ is inclusive of the value and ( is exclusive of the value for the bins in the *x*-axis as defined in standard mathematical notation, (**d**) surface complexity and correlation of ion intensities with TPS residuals for corresponding ions

We used a thin plate spline (TPS) procedure to model spatial variation of py-MBMS ions in the field trial. Because of the randomized block design, genotypic effects should be randomly distributed throughout the field, so fitted values from this analysis represent environmental variation, while residuals represent genotypic effects plus error. The fitted values of the TPS models for the 421 ions displayed two distinctive patterns: simple (Fig. 1a) vs. complex surfaces (Fig. 1b). The surface complexity (SC) parameter was able to discriminate between these patterns, with values for simple surfaces close to 0 and values above 1 for complex surfaces. Among the 421 ions, 198 ions with null SC were free of microspatial influence (only corrected for “tray effects”), while the rest (*n* = 223 ions) were impacted to varying degrees (Fig. 1c). The correlation between the total ion chromatogram (TIC)-normalized ion intensities and the TPS residuals serves as another indication of the degree to which an ion is affected by microspatial variation (Fig. 1d). Fifteen out of the 17 ions used to quantify lignin in the spectra and all ions deriving from cell wall sugars and free phenolics had SC values in excess of 1, indicating that these cell wall components were affected by microenvironmental variation.

**Fig. 2:**
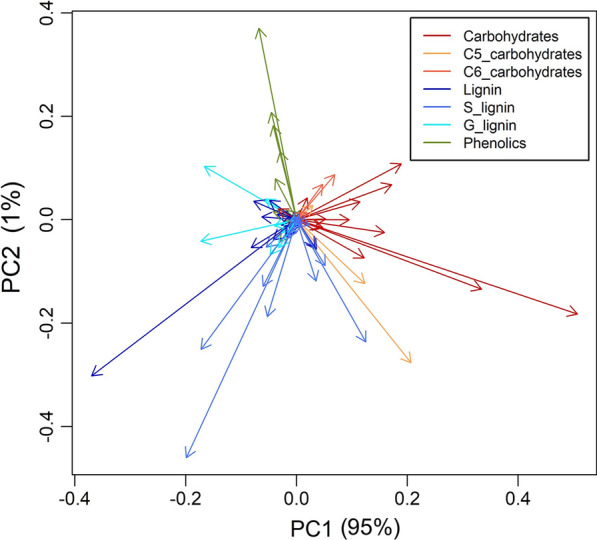
PCA loadings of thin plate spline (TPS)-predicted ion intensities from py-MBMS spectra of 7 × 7 population of *Populus trichocarpa* with sources of ions indicated

PCA of the ions based on their predicted TPS surface (Fig. 2), used here as a proxy for fine-scale environmental effects, yielded a PC-1 explaining 95% of the variation and PC-2 explaining 1%. The loadings for the first principal component were generally sugar-derived ions negatively correlated with lignin-derived ions with the exception of *m/z* 168 (primarily deriving from 4 to methylsyringol) and *m/z* 194. When ions of TPS-predicted surfaces were clustered in seven groups (Additional file 1: Fig. S5), the largest group (containing *m/z* 97) was related to phenolics and lignin-derived species, and the second largest (with *m/z* 82) mostly consisted of sugar-derived ions and lignin dimers, again showing that these cell wall components vary spatially with the microenvironment. The rest of the clusters were small and were mostly composed of irrelevant or otherwise unannotated peaks. Peaks that are termed here as irrelevant may include noise, fragments associated with more abundant species (i.e., loss of a proton) or ions that may have many or unknown sources.

### Inter-ramet variation captured in py-MBMS spectra

**Fig. 3:**
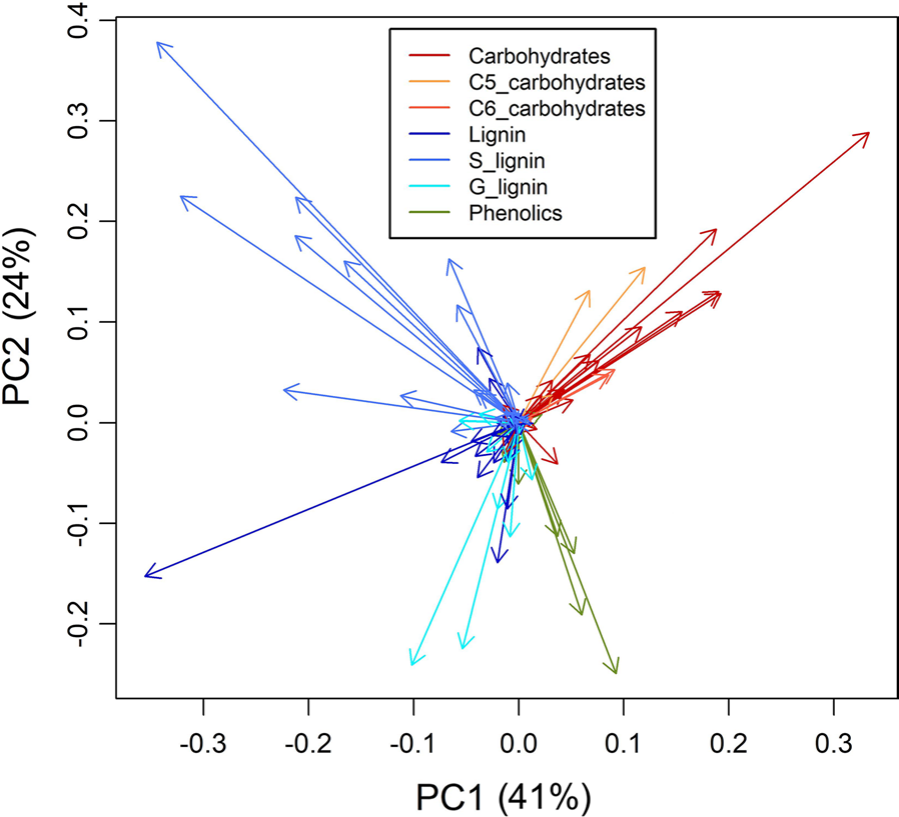
PCA loadings of TPS-corrected ions from py-MBMS spectra of 7 × 7 population of *P. trichocarpa*

**Fig. 4:**
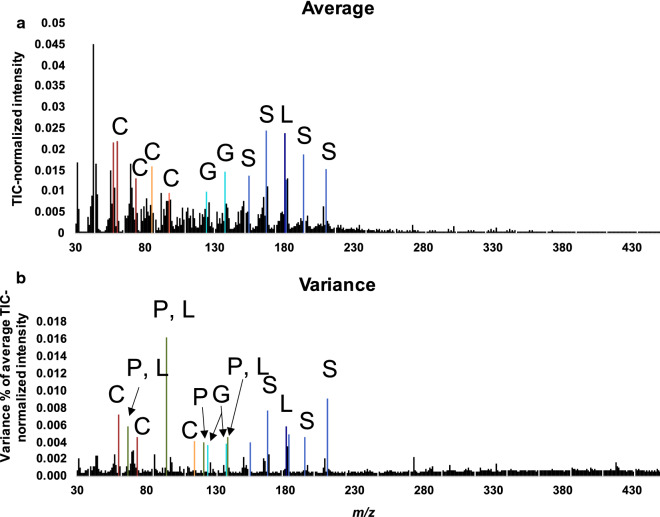
(**a**) Average spectrum of entire *Populus trichocarpa* 7 × 7 population after TPS and tray corrections and (**b**) variance (% of the average) of each ion based on TIC-normalized and corrected spectra. C denotes source as carbohydrate sugar, L: lignin, P: phenolics, S: S-lignin, G: G-lignin, colors correspond with those indicated in Figs. 2 and 3

After TIC normalization and controlling for instrument and environmental variation, the peaks derived from cell wall components had high loadings values in PCA (Fig. 3) and were also among the most abundant and had high variance relative to the mean intensities measured across the population as shown in Fig. 4a, b (Additional file 1: Fig. S6 shows PCA scores that are color coded corresponding to different field locations comparing before and after instrumental and environmental corrections). Approximately, 120 ions were annotated based on comparisons with standards, libraries and literature where unannotated ions are either representative of an unknown component or have many sources such that their presence or source is not included or discussed (see Additional file 2). The variance was highest for ions *m/z* 60 (C), 73 (C), 114 (C), 124 (G), 137 (G), 154 (S), 167 (S), 180 (L), 182 (S), 194 (S), and 210 (S) (where C denotes carbohydrate sugars, L for lignin, P for phenolics, G for G-lignin, S for S-lignin). These ions were also generally abundant in the spectra. However, other abundant ions such as *m/z* 57 (C), and 85 (C) did not exhibit as high variances relative to the mean intensity values as the former. Conversely, some ions were not particularly abundant but had high variance such as *m/z* 66 (P, L), 94 (P, L), 121 (P, L), and 138 (P, L).

PCA of the spatially corrected ions also revealed negative correlations in lignin-derived ions (e.g., *m/z* 124, 137, 154, 210) and carbohydrate-derived ions (e.g., *m/z* 73, 85, 114, 126) (Fig. 3). PC-1 accounted for 41% of the spectral variation, where carbohydrate-derived ions generally were negatively correlated with lignin-derived ions. PC-2 accounted for 24% of the spectral variation, with carbohydrate and syringyl (derived from sinapyl monomers, S) ions were negatively correlated with guaiacyl (derived from coniferyl monomers, G)-derived ions (Fig. 3). Additionally, *m/z* 66, 94, 121 and 138 were negatively correlated with other lignin-derived species, likely indicating these ions were primarily derived from phenolics (such as phenols occurring as secondary metabolites in the case of salicylates or other lignin-like, but not true-lignin phenolics such as ferulate, coumarate, etc.) as opposed to the fragmentation of lignin-derived pyrolysates (although a positive contribution from lignin-derived analytes cannot be ruled out).

### Heritability of py-MBMS spectral features

Gains in broad-sense heritability of the ions due to tray correction were marginal in most cases, though heritability of a few ions did improve noticeably with the correction (Additional file 1: Fig. S7). Values of broad-sense heritability for the TPS-corrected ion intensities ranged from 0 to 0.79, with annotated ions of highest heritability and noteworthiness summarized in Table 1. Permutation tests displayed thresholds of significance in heritabilities ranging from 0.028 to 0.037 for the combined tray-corrected and the TPS-corrected datasets. Although the ions with higher heritabilities were usually associated with complex surfaces for the TPS-fitted values, some ions with high heritability had simple TPS surfaces and SC values near 0 (e.g., *m/z* 55, 95, 167, 179, 181, 193, 195, 272, 312, and 302; Additional file 3).

**Table 1:** Most heritable and informative ions with corresponding sources observed in py-MBMS spectra of 7 × 7 *P. trichocarpa* pedigree

Ion (*m/z*)	Source	Heritability
94	Phenolics, lignin	0.79
138	Phenolics, G-lignin	0.76
66	Phenolics, lignin	0.74
121	Phenolics, lignin	0.74
167^a^	S-lignin	0.72
182	S-lignin	0.70
210	S-lignin	0.68
181^a^	S-lignin	0.65
194	S-lignin	0.64
154	S-lignin	0.59
208	S-lignin	0.58
124	G-lignin	0.56
150	G- and H-lignin, ferulate	0.55
164	G-lignin	0.53
137	G-lignin	0.53
168	S-lignin	0.48
180	Lignin	0.47
73	Carbohydrates	0.45
109	Phenolics, lignin	0.45
60	Acetyl	0.45
114	C5 carbohydrates	0.44
272^a^	G–G lignin dimer	0.41
286	Lignin dimer	0.38
151	G-lignin	0.36
57	Carbohydrates	0.37
126	C6 carbohydrates	0.32
98	C6 carbohydrates	0.32
85	C5 carbohydrates	0.31

Sources obtained based on analysis of standards and from comparison to NIST online databases and also from [9, 18, 27]

^a^Denotes surface complexity < 1

The most heritable ions (Table 1) also exhibited high variance in the population. However, several ions, including *m/z* 126 (C6 carbohydrates), 150 (G and H-lignin), 164 (G), 168 (S) 109 (P, L), 286 (G dimer) and 98 (C6 carbohydrates) were amongst the most heritable but exhibited relatively low variance. Maternal influence was almost always stronger for the most heritable ions, particularly lignin and phenolic-derived species. However, paternal effects were either more dominant or similarly influential as maternal effects for ions derived from carbohydrate sugars such as *m/z* 73, 97, 114 (see Additional file 3 for full comparison of paternal and maternal variance associated with each ion and Additional file 1: Fig. S8 for the % variance explained by mother vs father annotated by ion origin in biomass).

Hierarchical clustering (HC) using Spearman’s rank correlation distance metric with the complete linkage criterion was used to analyze the clustering of ions in combined tray–TPS-corrected spectra to elucidate spectral associations based mostly on genetic information. Eight groups were elucidated in the spectra based on K-means clustering (Additional file 1: Fig. S9), summarized in Table 2 (full spectral groups outlined in Additional file 4). Groups separated based on biocomponent sources similarly as those in the only TPS-corrected ions (Additional file 1: Fig. S10), indicating the majority of ions impacted by microspatial environment, and not ions highly impacted by instrumental variation, also were impacted by genetic variation of the population. Ions in the complete tray–TPS-corrected spectra generally clustered according to biopolymer source although unannotated and noise ions appeared in all clusters to some degree. Interestingly, the most heritable ions (*m/z* 66, 94, 121, 138), which are produced from phenolics (possibly including salicylate-like metabolites known to occur in *Populus* [35–39]), were clustered together in cluster *EK0* along with some lignin-derived species, including lignin dimers (*m/z* 272, 286). The rest of the most heritable ions clustered according to their biocomponent source in clusters *EK4* (G-lignin), *EK5* (carbohydrate sugars), and *EK6* (S-lignin) (Additional file 4).

**Table 2:** Classes of clusters of ions (complete spectra of thin plate spline and tray-corrected ions, clustered by Spearman’s rank correlation complete linkage hierarchical clustering) annotated for cell wall composition relevance

Cluster class (EK#)	Number of ions	Annotations
0	79	Phenolics, lignin dimers
1	103	Lignin dimers, large mass noise
2	40	Low and moderate mass noise
3	32	Low and high mass noise
4	58	G-lignin
5	50	Carbohydrates
6	34	S-lignin
7	25	Lignin, carbohydrate, and phenolic fragments, moderate mass noise

### Familial patterns of ramets

**Fig. 5:**
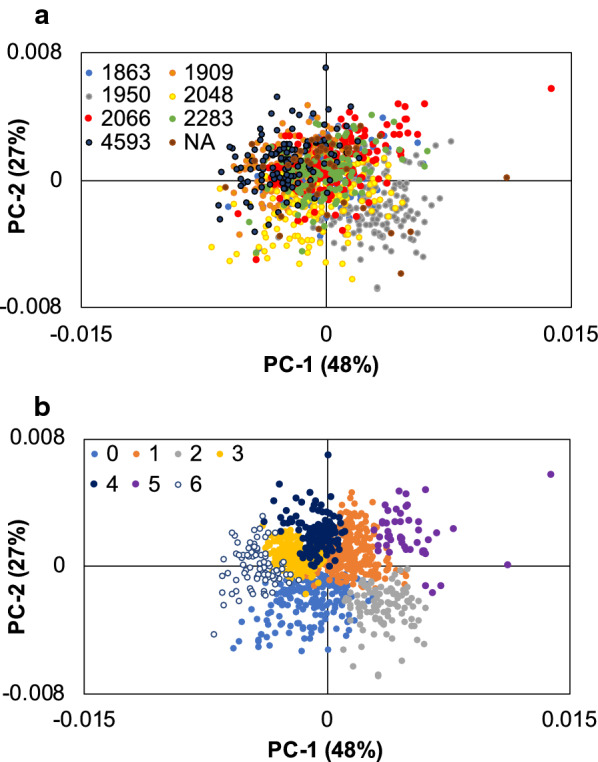
(**a**) PCA of clonal-averaged spectra with maternal/female parent genotype ID indicated (NA indicates maternal parent identity not available) and (**b**) Ward’s method cluster number indicated

Clustering of the samples based on the genotypic predicted values for py-MBMS spectra revealed some of the underlying family structure present in this population. PCA shows some differentiation of maternal half-sib families (Fig. 5a). The half-sib family from female/maternal ID 1950 (See Additional file 1: Table S6 for additional identifier information for each parent) in the lower right quadrant of the PCA scores plot had lower S/G and lower lignin composition in comparison to the half-sib family from female 4593 in the upper left quadrant of the plot (also see Additional file 1: Fig. S11). Clustering by Ward’s method using Euclidean distance revealed 7 clusters (Additional file 1: Fig. S12), where samples were previously classified into these groups based on K-means clustering meant to elucidate at least 7 different families (Additional file 1: Fig. S13). These groups largely corresponded to the maternal half-sib families (clusters colored in Fig. 5) as opposed to paternal half-sib families. Interestingly, one group of siblings (from female/maternal ID 1950) also produced the highest abundance of ion *m/z* 94 (PCA color coded in Additional file 1: Fig. S14), which can come from lignin but is otherwise attributed to the presence of phenolics such as salicylates (and in this case is otherwise not correlated positively with other lignin ion abundances such as *m/z* 210 as described previously (Additional file 1: Fig. S15 for example). The clustering of the samples in conjunction with PCA of the spectra shows that compositional relationships of several families can be elucidated, particularly based on the abundance of lignin and phenolic species. In some cases, several families generally produced similar or overlapping spectral features and, hence, appeared to have similar biomass composition, thus differences were not noted. Additionally, clustering methods differentiating PCA projections or scores based on MBMS spectra may identify spectral groups that consist of many family types and members, and do not necessarily separate families based on spectra. Either way, the use of clustering methods in combination with PCA of the py-MBMS spectra enables the visualization and validation of the compositional relationships within or across families that are able to be captured in the spectra.

### Analysis and heritability of cell wall traits from corrected py-MBMS spectra

**Fig. 6:**
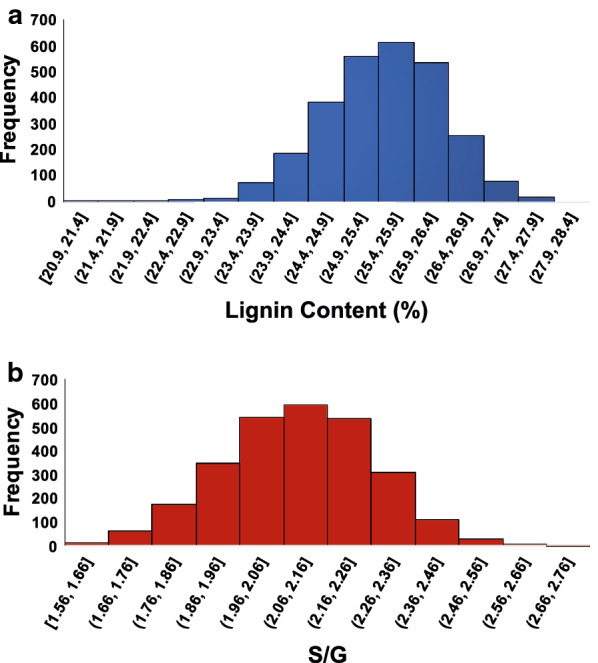
Histograms of lignin content and S/G ratios for the *Populus trichocarpa* population for all participants after thin plate spline correction for microspatial variation. [ is inclusive of the value and ( is exclusive of the value for the bins in the x-axis as defined in standard mathematical notation

The average lignin content of the entire population (*n* = 2721), after correcting for microspatial variation of the genotypes, was 25.5% (after taking replicate averages for each sample into account), ranging from 20.9 to 27.9% (Table 3; Fig. 6a). The S/G ratio ranged from 1.56 to 2.77, with an average of 2.10 (Table 3; Fig. 6b). These lignin metrics are typical for variants of *P. trichocarpa* as previously determined in Muchero et al. [1]. The broad-sense heritability of lignin composition based on TPS-corrected values was 0.56 and the heritability of S/G was 0.81.

**Table 3:** Summary of composition analysis for *Populus trichocarpa* pedigree samples based on thin plate spline corrected data, parenthesis indicate standard deviation

*Populus* family analysis	Population (*n* = 2721)	Heritability (*H*^2^)
Average lignin content (%)	25.5 (± 0.9)	0.56
Average S/G ratio	2.10 (± 0.17)	0.81

## Discussion

### Instrumental variance of py-MBMS spectral features

Variance associated with sample heterogeneity and changes within the analytical equipment (instrumental drift) are assumed to be the source of the variance that was removed by correcting for “tray effects.” Ions that were highly affected by the “tray variable” were mostly fragment ions typically related to a more abundant parent pyrolysate and therefore suspected to be more affected by the conditions of the instrumentation. Tray corrected ions consisted primarily of ions > 200 *m/z* which are generally unannotated, low abundance ions with many typically considered “noise.” Also, a significant amount of the ions, particularly those < 200 *m/z*, with high instrumental variance were also odd-numbered (as opposed to even), indicating their primary source as a fragment ion. For example, *m/z* 194 is known to originate from 2,6-dimethoxy-4-(2-propenyl) phenol, an abundant pyrolysate derived from a sinapyl moiety in the lignin polymer [9, 27] did not need correction based on “tray effects” but had significant effects from microspatial environment. Ion 193, being related to 194 after loss of a proton, had large tray effects and low associations with the microspatial environment (Additional files 2, 3, 4).

### Genetic and environmental influences on py-MBMS spectral features

Microspatial and genetic variance component analyses on the MBMS data separated the ions into several distinct groups, of which the following were noted: (i) ions not affected by microspatial environmental variation with no significant heritability, (ii) ions not affected by microspatial environmental variation with high heritability, (iii) ions slightly affected by microspatial environmental variation with high heritability and (iv) ions moderately affected by microspatial environmental variation with moderate heritability, where most of the ions present in groups i–iii were tray-corrected as described in the previous section. Ions present in group i could mostly be attributed to “noise” and are otherwise not important for trait analyses and breeding approaches. The ions with high heritability but not affected by fine-scale environment (group ii) consisted mostly of ions that fragment from other highly heritable ions, including ions 181 (being related to 180 and 182) and 167 (an ion produced from a wide variety of S-lignin-derived species [9, 27] that are otherwise also heritable). The heritable and environmentally stable ions still need to be corrected for instrumental drift as they may be particularly sensitive to the conditions of the pyrolyzer impacting fragmentation (e.g., the “parent” ions such as 182 and 194 would have environmental correction and subsequent fragments 181 and 167 (respectively) would require instrumental correction). Otherwise, some peaks that were not impacted by microspatial environment but were impacted by instrumental variance and were highly heritable are noteworthy based on their roles in lignin structure. For example, *m/z* 272 and 302, believed to originate from G–G and S–G dimers in lignin [27], were heritable after tray correction but did not have high impact from environmental conditions, indicating low phenotypic plasticity for these traits. The 223 ions that were affected by microspatial environment (groups iii and iv), and subsequently TPS-corrected, included most of the ions known to originate from cell wall polymers including cellulose (e.g. *m/z* 126), hemicelluloses (e.g., *m/z* 114) and lignin (such as *m/z* 124, 154, 168, 208) all together primarily occurring between *m/z* 50 and 210 (see Additional file 2 for annotations). Therefore, ions impacted by the environment originating from the cell wall components were necessarily corrected for accurate heritability measurements and exhibited high phenotypic plasticity. After the microspatial environmental and instrumental corrections for the ions in the spectra were made, the genotypic variation associated with the fragmented ions is more likely the result of variation in the structure of the cell wall biopolymers (i.e., lignin linkages) and presence of free phenolic metabolites. However, more highly resolved information would be necessary to derive the specific biopolymer differences in heritable spectral features due to the complicated processes associated with thermal decomposition and fragmentation of bio-derived species during pyrolysis and electron ionization.

Clustering of the ions on the TPS-predicted values identified groups of ions with similar responses to microspatial variation in the field trial. The two largest groups that represented the principal components of the TPS-predicted values primarily corresponded to lignin and phenolic-derived ions (*FK1*) and lignin dimer as well as sugar-derived ions (*FK6*). The patterns of spatial variation in predicted values (e.g., Fig. 1b) roughly corresponds to areas of the field that had high water content and standing water during the winter (personal observation). PC-1, accounting for 95% of the variation in the data, may therefore reflect this variation in soil water content. Therefore, most ions were affected by this single environmental factor, but in opposing directions: FK1 ions (phenolics, G- and S-lignin) had lower values in the areas under excess water, whereas FK6 (sugar and lignin dimers) had higher values with excess water. The other five clusters were driven by other minor PCs, and no clear pattern could be inferred. Independent clustering on TPS-corrected values resulted in groups that were largely congruent with those identified by the TPS-predicted values. However, clustering of ions based on the TPS-corrected values should be driven by intrinsic factors of each ramet, including genetics, and was hence more effective at resolving genotypes on the basis of lignin monomer composition. Thus, ions in the same cluster are likely to share some of the same underlying genetic control, and may be derived from related pathways. This may provide a valuable clue about the identities of some of the unknown ions in these clusters warranting further investigation on their sources.

### Sources of ions that were highly heritable and impacted by microspatial variation

Hierarchical clustering of the ions showed groups of ions relating to certain cell wall components and Ward’s clustering of the samples was able to differentiate the samples based on familial relationships that were related by corresponding cell wall compositional differences. Lignin-derived ions were highly heritable, particularly those originating from the sinapyl (S) monomer present in the lignin polymer. For example, *m/z* 210, 194, 182, 167, 154 are all ions present in the spectra of sinapyl alcohol consisting of fragments corresponding to 2,6-dimethoxy-4-(2-propenyl)phenol (*m/z* 194), 4-ethylsyringol (*m/z* 167), and syringol (*m/z* 154), each of which are subsequently produced from various syringyl monomers present in lignin [40, 41]. The ion *m/z* 208 originates from sinapylaldehyde which can be generated upon pyrolysis of S-lignins but if present in native lignin, can have dramatic implications relating to genetic and recalcitrance properties [42]. Ions 182 and 181 are due to the presence of syringaldehyde, which can be produced upon pyrolysis of syringyl lignin but may also be present in native lignin structures [42]. Ions 124 and 137 derive from a great number of guaicyl moieties (from coniferyl, G, monomer) in the lignin polymer, whereas 164 derives primarily from eugenol which was likely produced upon pyrolysis of labile (i.e., β-*O*-4) guaiacyl units in the lignin [43]. Other guaiacyl-related ions consisted of *m/z* 151 and 152, indicating the presence of vanillins which are primarily produced from the pyrolysis of guaiacyl lignins and ferulates but may also be present in the native lignin polymers [44]. Ferulic acid and 4-vinylguaiacol are likely the sources of *m/z* 150 and 135. There are many S- and G-derived pyrolysates attributing to the presence of *m/z* 168 and 180, hence their high abundance in the spectra and high variance. Interestingly, *m/z* 272 is likely derived from a guaiacyl stilbene dimer that has been observed from the pyrolysis of lignins [27, 43] but has recently been identified as a monomer in endocarp lignins [45]. Additionally, since the samples were analyzed without an extraction of low-molecular weight phenolics, the heritability of phenolic-derived ions and the relationship between phenolic- and lignin-derived ions was observable. One set of half-siblings in particular, exhibited low S/G and low lignin abundance with higher production of phenolic/salicylate-derived ions.

The most heritable and most variable ions originating from sugars were generated from many sugar-derived species including *m/z* 60 (derived from hydroxyacetaldehyde and acetate) and 73 (3-hydroxypropionaldehyde), each of which are also fragments of levoglucosan produced upon the pyrolysis of sugars [27]. Otherwise, *m/z* 114 which is particularly produced from C5 sugars (i.e., xylose [24, 27]), was amongst the most heritable carbohydrate-derived ions. C6 sugars (i.e., glucose from cellulose [24, 27]) are known to produce *m/z* 126, which was also found to be heritable but to a lesser degree than the C5-derived *m/z* 114. These sugar-derived ions were driven similarly by both maternal and paternal effects, but were amongst those of highest variance from paternal effects.

### Maternal effects

Ions originating from phenolics and salicylate-like species (and as fragments from lignin), including *m/z* 66 (P, L), 94 (P, L), 121 (P), and 138 (P, L), were primarily driven by maternal parent-of-origin effects and these were among the most heritable as well as the most variable ions produced from the population.

Parent-of-origin effects are when offspring phenotypes are not in line with an additive pattern of inheritance of the two progenitors where deviation is mostly biased to either the female progenitors or to the male progenitors. For example, chill tolerance in cucumber (*Cucumis sativus*) displays strong maternal effects (when the phenotype of the mother prevails over the father [46]) and seems to be linked to chloroplast elements [47, 48]. Parent-of-origin effects have been shown to have important ecological and evolutionary implications (for example, genomic imprinting where the silencing or overexpression of alleles is based on the sex of parent of origin, review provided by [49]). Given the asymmetric dispersal distance between ovules (maternal) and pollen (paternal), maternal effects seem to play a role in local adaptation of offspring to environments that normally are more similar to that of the mother than the father [50]. Also, parent-of-origin effects have been implicated in interspecific reproductive barriers [51, 52] with a high rate of diversifying evolution among plant species through genomic imprinting [53]. However, imprinted expression is conserved in other genes across plant lineages, suggesting stabilizing selection for these loci [53, 54].

Despite the apparent evolutionary and ecological importance of parent-of-origin effects and their common occurrence in quantitative genetics studies of many traits of diverse plants [55], further investigation of causality is lacking. Furthermore, most in-depth studies have focused on the early stage of plant life cycle (endosperm and seed development [46, 56, 57]) and not much is known about genomic-imprinting and organelle effects in later stages of life in plants. In this study we show how parent-of-origin effects (mostly maternal) have an important role in wood chemistry profiles. This may suggest that wood composition could play important roles in environmental adaptation in plants.

### Implications in breeding and genomic selection

The high level of accuracy and reproducibility of py-MBMS instrumentation showcased by this experiment and the high heritability of many biologically relevant ions provide many possibilities for the use of this analytical technique in tree breeding. First, precise and rapid estimation of lignin content and S/G ratio allowed for large sample size (i.e., breeding population or more replication) and the reduction of error, that translate immediately to gains in selection efficiency. The broad-sense heritability values observed in this study for total lignin (0.56) and S/G (0.81) were comparable to or higher than those observed in previous studies using similar methodology (range of 0.23–0.58 and 0.42–0.81 for lignin and S/G, respectively; Additional file 1: Table S7) [3, 58–60]. Second, this method and data treatment also permits larger training populations to create models for genomic selection with a lower level of uncertainty. Finally, recent research has pointed towards the integration of phenomics layers into the genomic selection process to increase predictive power of the models [61], making use of the genetic correlation between phenotypes and also being able to improve associations between the genome and the traits. This latter approach takes advantage of the normally simpler genetic architecture of some phenotypes (such as specific pyrolysates or metabolites) to detect true positive associated loci. In this sense, to select for a trait, other layers of phenotypes are added to help to establish true correlations between the genome and the phenotype of interest. Among the many approaches that are under investigation currently, the use of selection indices seems promising [62]. Selection index approaches could be used not only to select for several phenotypes simultaneously, but also to integrate other phenotypes in the selection process that are not of ultimate interest but due to high correlation with the phenotype of interest and high heritability can improve accuracy substantially.

## Conclusions

Comprehensive analysis of a large pedigree of *Populus trichocarpa* was streamlined in a high-throughput py-MBMS analysis and data analytics framework that elucidated variation and heritability of biomass composition on the basis of spectral features. Ions that were sensitive to microspatial variation originated from phenolics and cell wall biopolymers such as lignin and hemicelluloses and cellulose; whereas, ions that were primarily impacted by instrumental variance were primarily attributed to noise, fragments and unannotated ions. The most variable and heritable ions originated from phenolics and S-derived lignin monomers with fewer highly heritable ions originating from G-derived monomers and carbohydrates within the cell wall. Maternal effects were generally higher for most heritable ions, particularly those derived from phenolics and lignin; whereas, paternal effects were similar to maternal or more impactful for carbohydrate-derived ions.

## Methods

### Progeny trial design and sample collection

Seven females and seven males of black cottonwood (*Populus trichocarpa*) were collected from naturally occurring stands in Oregon and Washington, USA and were selected based on extremes and intermediate values in lignin content and S/G ratio phenotypes. These trees were grown in a common garden in Westport, OR on the lower Columbia River. These trees were crossed to generate 49 full-sib families in a full factorial design (i.e., full factorial; 7 × 7), parent identifier information is provided in Additional file 1: Table S6. Additionally, seven open-pollinated families were created from the seven mothers. In total, 986 offspring were obtained and propagated. Three clonal replicates of these progeny plus the 14 parents (*n* = 1000) were planted in April of 2015 in three complete, randomized blocks in Westport, OR. The trial consisted of 30 rows with 100 trees per row and the trial was surrounded by a double border consisting of extra ramets from the same crosses (Additional file 1: Fig. S16). Spacing was 3 m between rows and 1 m within rows. The location of the trial (46.130742°N, 123.370276°W) falls within the core of the natural black cottonwood distribution, with favorable climatic and edaphic conditions for this species.

In January 2018, wood cores were collected at breast height (1.3 m) from the southern face of the trunks of all live ramets (*n* = 2721). The cores were stored in envelopes and dried at 50 °C for 72 h. They were then ground in a Wiley Mini Cutting Mill (Thomas Scientific) and filtered through a 20 mesh sieve to 1 mm for py-MBMS analysis.

### Py-MBMS experiments

Py-MBMS was performed according to the previously described methods [9, 18, 23, 63]. Briefly, 4 mg of debarked, dried and ground material (1 mm mesh) was pyrolyzed in a Frontier PY2020 at 500 °C for 30 s. Pyrolysis vapors were analyzed using an Extrel Super Sonic MBMS Model Max 1000 and spectra were collected at 17 eV from *m/z* 30–450. MBMS data were processed using Merlin 3.0 software to produce average spectrum acquired during sample pyrolysis (a total ion chromatogram (TIC) peak over ~ 0.5 min at 0.5 s/scan rate) and spectral analysis was performed on (TIC) sum-normalized data as described in the following sections. Almost all samples of ground wood were run twice (*n* = 5440). Additionally, six standards were run throughout (*n* = 509) for quality control monitoring. Runs were carried out in a total of 125 forty-eight-vial trays. Samples were analyzed in one replicate prior to instrument cleaning and retuning and the second replicate (both sets in random order) was collected after cleaning to validate the reproducibility of the spectra. Lignin content was estimated by summing *m/z* 120 (H, coumarate), 124 (G), 137 (G), 138 (G), 150 (H, G, ferulate), 152, 154 (S), 164 (G), 167 (S), 168 (S), 178 (G), 180, 181, 182 (S), 194 (S), 208 (S) and 210 (S), where H denotes coumaryl (H) monomer-derived species, G refers to species derived from coniferyl monomers and S derived from sinapyl monomers. S/G was determined by dividing sum of S-derived ions (154, 167, 168, 182, 194, 208, 210) by the sum of G-derived ions (124, 137, 138, 150, 164, 178). Coumaryl (H) content was not determined based on known overlaps with coumarate-derived ions. Ion annotations were made based on [9, 18, 24, 27], comparisons to NIST databases (online as there is not one available for MBMS software) and by comparisons to standards analyzed in-house (spectral information available upon request).

### Correction of py-MBMS instrumental drift (tray effect)

This uniquely large sample set from a population developed for quantitative genetic analyses required the development of a data analysis workflow that encompassed QC and comprehensive spectral analysis. To assess and correct for the batch effect due to tray, the following linear mixed model was run for every single ion for the six replicated standards:

$$y_{i,t,s}= \mu +{\rm Tray}_{t}+{\rm Standard}_{s}+{\varepsilon}_{i,t,s},$$

where, *y*_*i,s,t*_ is the TIC-normalized intensity of the ion *i* in the tray *t* for the standard *s*, *μ* is the overall mean, Tray_*t*_ is the tray, Standard_*s*_ is the identity of the standard and Ɛ_*i,t,s*_ is the error. Both tray and standards were treated as random effects. The effect of each tray was used to correct the spectrum reads of each ion in the whole dataset (henceforth tray-corrected dataset). For one tray that lacked standards, a model was run on the whole dataset just having tray as random effect. These models were solved with R package *lme4*.

### Thin plate spline model for microspatial variation analysis and correction of py-MBMS spectra

The means of each ramet of the spectral values for each ion were regressed to their spatial position in the field trial using a Thin plate spline model (TPS). This model estimates a smoothed surface, in our case matching with the two-dimensional spatial layout of the trial, but minimizing the variance of the residuals. Therefore, the predicted values of the model serve as an estimation of how the fine-scale environmental variation affects the ion intensity value, and the residuals quantify the variation that is attributed to other factors, such as genotype. Thus, we used the model to: (i) pinpoint which ions were not affected by fine-scale environment; those ions could be putative noise (i.e., no biological significance), metabolites under very strong genetic control, or stochastic or hyper-responsive metabolites, (ii) cluster ions based on their response to the environment and (iii) correct for the effects of fine-scale environmental variation. When the TPS model of an ion predicts a simple surface, it implies that the ion is not affected by fine-scale environmental variation (Fig. 1a). Complete lack of association between environment and phenotype is unlikely. Therefore, this criterion could be used as a line of evidence to identify noise. However, linkage between environment and phenotype could be obscured for metabolites that are hyper-responsive to the environment, or which vary stochastically. Finally, metabolites under strong genetic control would not be expected to vary spatially. The complexity of the TPS-fitted values was assessed with a ad hoc parameter, dubbed as Surface complexity (SC), employing the following formula:

$$\mathrm{SC}= -{\mathrm{log}}_{10}\prod_{i}^{I}\left|\mathrm{cor}({\widehat{y}}_{i},t)\right|,$$

where, *i* is the *i*-th row, *I* is the total number of rows, $\widehat{{y}_{i}}$ is the vector of the fitted values for this row *t* is the vector of the tree position in the row, and cor is the Pearson correlation coefficient. Predicted values in simple surfaces vary linearly within row, so the absolute value of the correlation between the predicted value and the tree position within row is almost one. The product of this correlation across all the rows should therefore be close to one, and the log of this value should approach zero. Conversely, the higher complexity the predicted surface has, the lower the correlations will be and the higher the parameter SC will be. The R package *fields* [64] was used to fit the TPS models.

To group the ions based on response to the environment, the predicted values from the TPS model were submitted to a principal component analysis (PCA) and to hierarchical clustering (HC) as described below. Finally, the residuals of the models were used as estimators of the variation of the ions due to factors not spatially driven (e.g., genotype). The residuals for each were added to the ion mean value to project them to the original scale (henceforth TPS-corrected dataset). To study common non-environmental trends among ions, PCA on standardized values and HC were carried out as described below.

### Broad-sense heritability

Broad-sense heritabilities were calculated on the TIC-normalized, combined tray-corrected and the TPS-corrected full spectra and TPS-corrected values of lignin and S/G ratios determined prior to any spectral corrections. These variance components were estimated using a linear mixed model having ion intensities as response variable and genotype as a random effect by means of the R package *lme4*. Heritability was calculated from the variance explained by genotype divided by the total variance (i.e., the sum of the variance explained by genotype plus the variance due to residuals of the model). To assess significant departures from null values, we performed permutation tests on the heritability of each ion. We ran 1000 permutations for each ion setting significance threshold α ≤ 0.05. Maternal and paternal effects were estimated in a similar way, adding mother and father as random effects to the aforementioned model. For example, maternal effects were estimated by the proportion of the variance explained by the mother relative to the total variance (sum of variance explained by genotype, mother, father and residuals).

### Multivariate analytical procedures

Descriptive statistical analysis (i.e., mean, variance, standard deviation), principal component analysis (PCA) and clustering were used to explore spectral data with the software R [65] and Unscrambler X V.10.5 (Camo Software). PCA was performed using 100 iterations of the NIPALS algorithm, with 6 or 7 principal components depending on convergence of data, 20 random cross validation segments, and mean-centered data. Hierarchical clustering by complete linkage using Spearman’s rank correlation distance was performed for clustering of ions in spectra and Ward’s method using Euclidean distance for clustering of biomass samples. K-means clustering of ions and samples (ramets) was performed in R using the packages *cluster* and *factoextra*.

## Supplementary Information

**Additional file 1: Table S1. **Quality control metrics for validation and comparison of sample analysis before and after cleaning on the basis of Aspen controls. Parentheses indicate standard deviation. Table S2. Summary of quality control composition metrics determined for P. trichocarpa pedigree samples. Parentheses indicate standard deviation. Values are determined prior to TPS and tray correction. *Two samples were analyzed once. Table S3. Annotations of ions in py-MBMS spectra (supplied as separate file). Table S4. Summary of variance, maternal and paternal effects of each ion after TPS and tray correction (supplied as separate file). Table S5. Comprehensive list of ions in 8 clusters from HC-SRC (supplied as separate file). Table S6. Additional identifier information associated with parents of the P. trichocarpa population. Table S7. Estimates of broad sense heritability for studies in the Salicaceae that used py-MBMS to estimate total lignin and/or S/G ratios for species from the Salicaceae. Figure S1. PCA of standards analyzed throughout course of poplar analysis. Scores plots with numbers at each point indicate the tray (chronological sequence) number associated with particular sample. A) Entire scores plot of all standards, B) zoomed scores plot of region consisting primarily of Center for Bioenergy Innovation (CBI) poplar, Aspen and Poplar 068, C) scores of Loblolly Pine 6G1 standard, D) scores of NIST 8492 (Populus Deltoides), E) scores of Poplar 93968, F) corresponding loadings of spectra from standards only plotted in spectral format for PC-1 and G) loadings for PC-2 of the standard spectra only. “Pop” corresponds to poplar and “Lob” corresponds to Loblolly pine. Figure S2. Aspen Control analysis throughout course of experiment. A) average spectrum B) variance of each ion. Figure S3. PC-1 as a function of time using Control Aspen spectra (TICnormalized) over the course of analysis by py-MBMS. X-axis values correspond to tray number (chronological sequence) in which the aspen sample was analyzed. >120 trays were analyzed over the course of 6 weeks. Figure S4. PCA analyses of replicate 7x7 poplar sample analyses. Colors show replicates prior to tray and TPS correction. Figure S5. TPS-predicted spectra hierarchical clustering dendrogram where colors show separation of 7 clusters corresponding to K-means clusters (Figure S9). Figure S6. PCA of entire population with spatial location of sample highlighted prior to TPS-tray correction of spectra and after TPS-tray corrections. Figure S7. Broad-sense heritability of ions in py-MBMS spectra with and without tray (A) and TPS (B) correction. Figure S8. Variance of ions in py-MBMS spectra explained from maternal and paternal effects. Figure S9. K-means cluster analysis of ions in spectra from 7x7 pedigree. Figure S10. Hierarchical Clustering (complete linkage, Spearman’s rank correlation distance method) of ions from spectra of entire population after tray and TPS correction. EK# shown corresponding to different colors of ions in dendrogram corresponding with Additional file 4 . Figure S11. Lignin content and composition differences based on maternal and paternal relationships. Figure S12. Clusters of samples after tray and TPS correction (Ward’s Euclidean, 7 clusters). Figure S13. K-means clustering of samples based on spectra after clonal averaging from 7x7 P. trichocarpa pedigree. Figure S14. PCA projection of corrected spectra from population after clonal averaging showing clustering associated with m/z 94 abundance (blue square being lowest, red circle is moderate and green triangle is highest intensity of m/z 94). Figure S15. Comparison of phenolic (m/z 94) and S lignin (m/z 210) derived ions across half-sib families, error shows standard deviation within each family. Figure S16. Block diagram of the progeny field design.

**Additional file 2:** Annotations of ions in py-MBMS spectra

**Additional file 3:** Summary of variance, maternal and paternal effects of each ion after TPS and tray correction

**Additional file 4:** Comprehensive list of ions in 8 clusters from HC-SRC

## Data Availability

The datasets used and/or analyzed during the current study are available from the corresponding author on reasonable request.

## Abbreviations

C6: Carbohydrates with 6 carbon sugars
C5: Carbohydrates with 5 carbon sugars
HC: Hierarchical clustering
PCA: Principle component analysis
py-MBMS: Pyrolysis-molecular beam mass spectrometry
SC: Surface complexity
S/G: Syringyl/guaiacyl ratio
TPS: Thin plate spline
