# Supplementary material for: Accurate determination of genotypic variance of cell wall characteristics of a Populus trichocarpa pedigree using high-throughput pyrolysis-molecular beam mass spectrometry
Source: Biotechnol Biofuels. 2021 Mar 6;14:59. doi: 10.1186/s13068-021-01908-y (PMC7937246; doi:10.1186/s13068-021-01908-y)

Supplementary Material

Results and Discussion

QC of MBMS spectra

After cleaning and retuning the equipment, replicate analysis of the 7x7 population was performed and validation of the original spectral pattern and estimated lignin contents and S/G ratios were established on the basis of correlative patterns and relative lignin content measurements. The spectral patterns captured in the loadings for PC1 and PC2 of Aspen control did not indicate correlative changes in the relative abundance of particular biopolymers over time although ions originating from S-lignin and carbohydrates were the largest source of spectral variance. The estimated lignin content of the Aspen control was not statistically different between the first group of replicates and the second replicate obtained after cleaning ($p = 0.1209$, Supplementary Table 1) prior to tray correction. Although the average S/G ratio before cleaning was lower than that observed after ($p < 0.0001$), this value is not corrected to a standard and is a relative estimated measurement that must be taken in context of other samples being analyzed (variance associated with instrumental drift). For validation, the Pearson correlation of S/G ratios of the poplar sample replicates was 0.92 ($R^2 = 0.86$), indicating that while the values determined for the second replicate were slightly higher as demonstrated in the control sample, the trends were highly correlative.

Supplementary Table 1. Quality control metrics for validation and comparison of sample analysis before and after cleaning on the basis of Aspen controls. Parentheses indicate standard deviation.

Aspen analysis	First replicate (n=62)	Second replicate (n=65)
Lignin content (%)	21.8 (± 0.4)	21.7 (± 0.3)
S/G ratio	1.72 (± 0.04)	1.77 (± 0.04)

Supplementary Table 2. Summary of quality control composition metrics determined for *P. trichocarpa* pedigree samples. Parentheses indicate standard deviation. Values are determined prior to TPS and tray correction. *Two samples were analyzed once.

Populus family analysis	First replicate (n=2719)*	Second Replicate (n=2721)	Average (n = 5440)	Pearson correlation between reps
Average corrected lignin content (%)	25.4 (± 1.0)	25.6 (± 0.9)	25.5 (± 0.9)	0.87
Average S/G ratio	2.08 (± 0.18)	2.11 (± 0.18)	2.10 (± 0.18)	0.92

Supplementary Table 3. Annotations of ions in py-MBMS spectra (supplied as separate file).

Supplementary Table 4. Summary of variance, maternal and paternal effects of each ion after TPS and tray correction (supplied as separate file).

Supplementary Table 5. Comprehensive list of ions in 8 clusters from HC-SRC (supplied as separate file).

Supplementary Table 6. Additional identifier information associated with parents of the *P. trichocarpa* population

SEX	CLONE	IDENTITY
Female	1863	GS-001-03
Female	1909	GS-006-04
Female	1950	GS-010-01
Female	2048	GS-017-09
Female	2066	GS-018-12
Female	2283	GS-033-05
Male	2365	GS-039-08
Male	2393	GS-040-10
Male	2515	GS-046-11
Male	2572	GS-050-08
Male	2683	GS-056-06
Female	4593	PS-52-90
Male	6909	GS-155-09
Male	7073	PS-33-94

Supplementary Table 7. Estimates of broad sense heritability for studies in the Salicaceae that used py-MBMS to estimate total lignin and/or S/G ratios for species from the Salicaceae.

Species	Lignin	S/G	Reference
P. trichocarpa	0.33	0.81	(Guerra et al., 2016)
P. nigra	0.58	0.70	(Guerra et al., 2013)
P. trichocarpa x P. deltoides	0.23	NA	(Zhang et al., 2014)
Salix viminalis	NA	0.42	(Ohlsson et al., 2019)

Guerra, F. P., Richards, J. H., Fiehn, O., Famula, R., Stanton, B. J., Shuren, R., Sykes, R., Davis, M. F., & Neale, D. B. (2016). Analysis of the genetic variation in growth, ecophysiology, and chemical and metabolomic composition of wood of Populus trichocarpa provenances. *Tree Genetics and Genomes*, *12*(1), 1–16. https://doi.org/10.1007/s11295-015-0965-8

Guerra, F. P., Wegrzyn, J. L., Sykes, R., Davis, M. F., Stanton, B. J., & Neale, D. B. (2013). Association genetics of chemical wood properties in black poplar (*Populus nigra*). *New Phytologist*, *197*(1), 162–176. https://doi.org/10.1111/nph.12003

Ohlsson, J. A., Hallingbäck, H. R., Jebrane, M., Harman-Ware, A. E., Shollenberger, T., Decker, S. R., Sandgren, M., & Rönnberg-Wästljung, A. C. (2019). Genetic variation of biomass recalcitrance in a natural Salix viminalis (L.) population. *Biotechnology for Biofuels*, *12*(1), 1–12. https://doi.org/10.1186/s13068-019-1479-7

Zhang, J., Novaes, E., Kirst, M., & Peter, G. (2014). Comparison of Pyrolysis Mass Spectrometry and Near Infrared Spectroscopy for Genetic Analysis of Lignocellulose Chemical Composition in Populus. *Forests*, *5*(3), 466–481. https://doi.org/10.3390/f5030466

Supplementary Figure 1. PCA of standards analyzed throughout course of poplar analysis. Scores plots with numbers at each point indicate the tray (chronological sequence) number associated with particular sample. A) Entire scores plot of all standards, B) zoomed scores plot of region consisting primarily of Center for Bioenergy Innovation (CBI) poplar, Aspen and Poplar 068, C) scores of Loblolly Pine 6G1 standard, D) scores of NIST 8492 (*Populus Deltoides*), E) scores of Poplar 93968, F) corresponding loadings of spectra from standards only plotted in spectral format for PC-1 and G) loadings for PC-2 of the standard spectra only. "Pop" corresponds to poplar and "Lob" corresponds to Loblolly pine.

A)

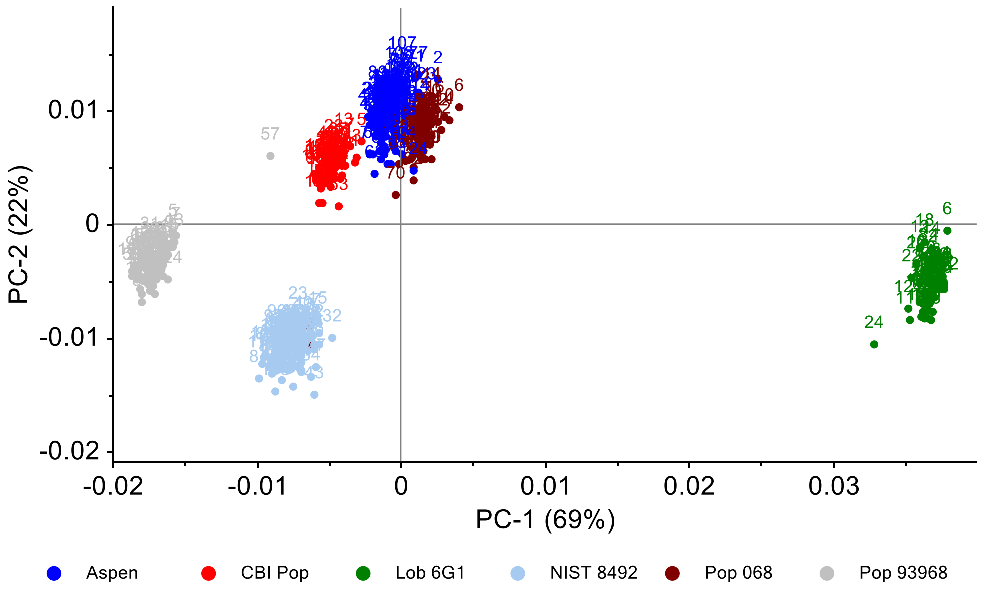

B)

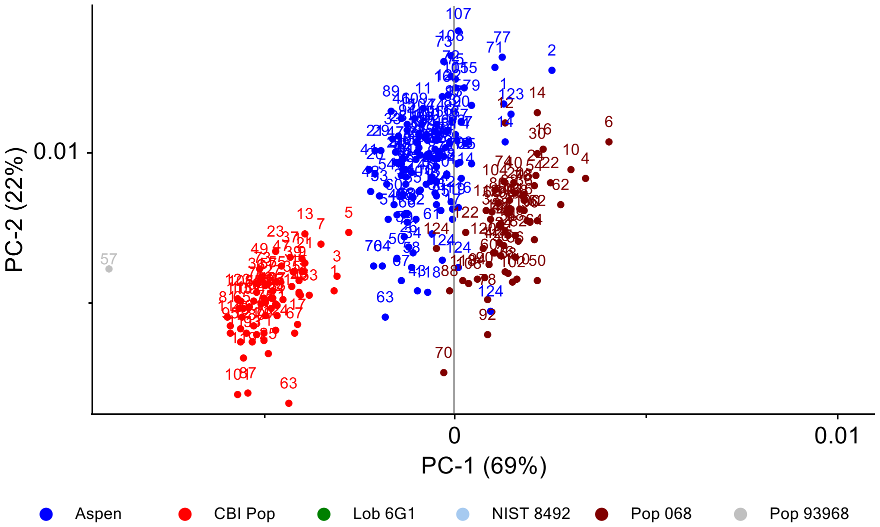

PC-2 (22%)
0.01
0
0.01
PC-1 (69%)
Aspen
CBI Pop
Lob 6G1
NIST 8492
Pop 068
Pop 93968

C)

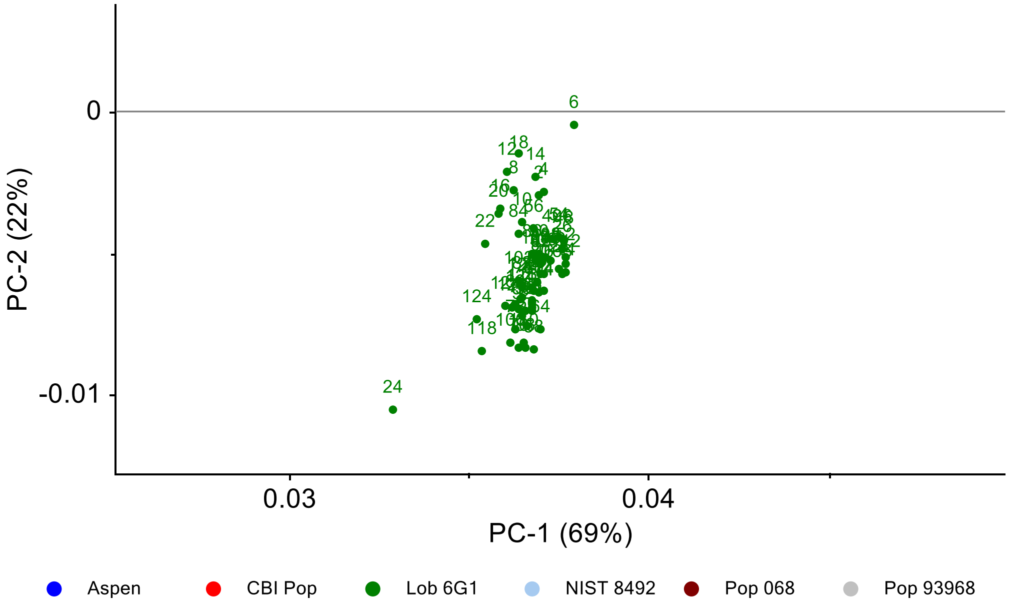

PC-2 (22%)
0
-0.01
0.03
0.04
PC-1 (69%)
Aspen
CBI Pop
Lob 6G1
NIST 8492
Pop 068
Pop 93968

D)

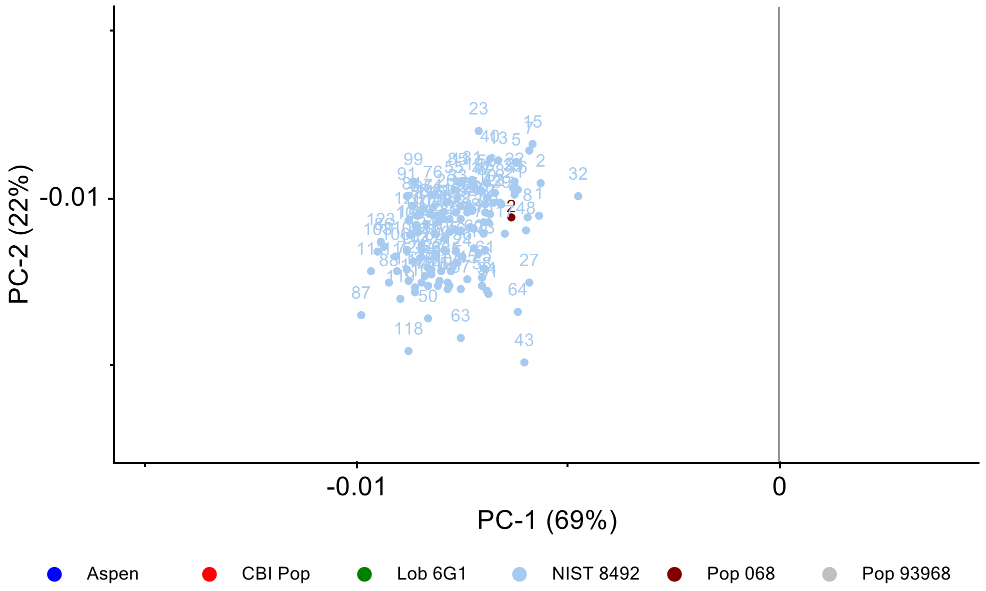
-0.01
PC-2 (22%)
-0.01
0
PC-1 (69%)
Aspen
CBI Pop
Lob 6G1
NIST 8492
Pop 068
Pop 93968

E)

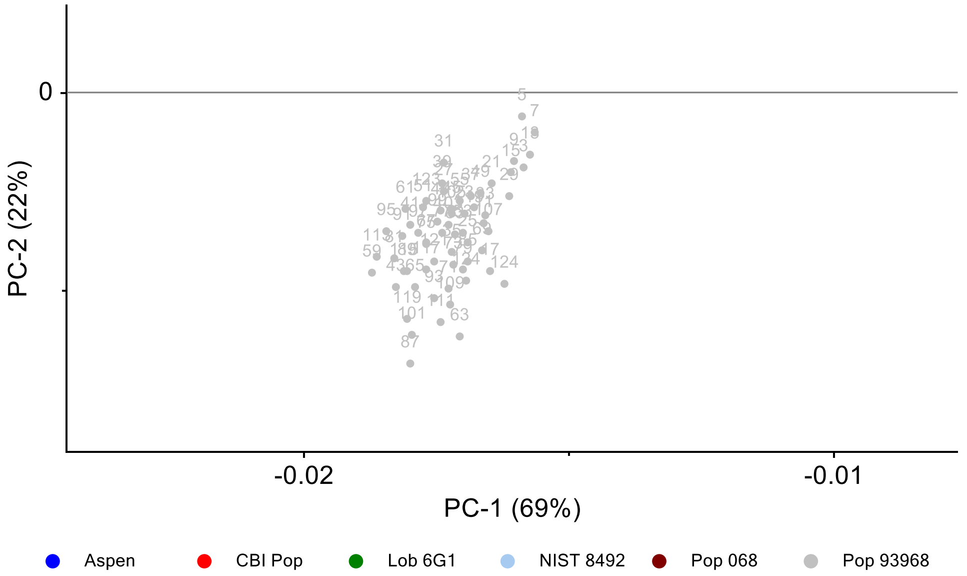
0
PC-2 (22%)
-0.02
-0.01
PC-1 (69%)
Aspen
CBI Pop
Lob 6G1
NIST 8492
Pop 068
Pop 93968

F)

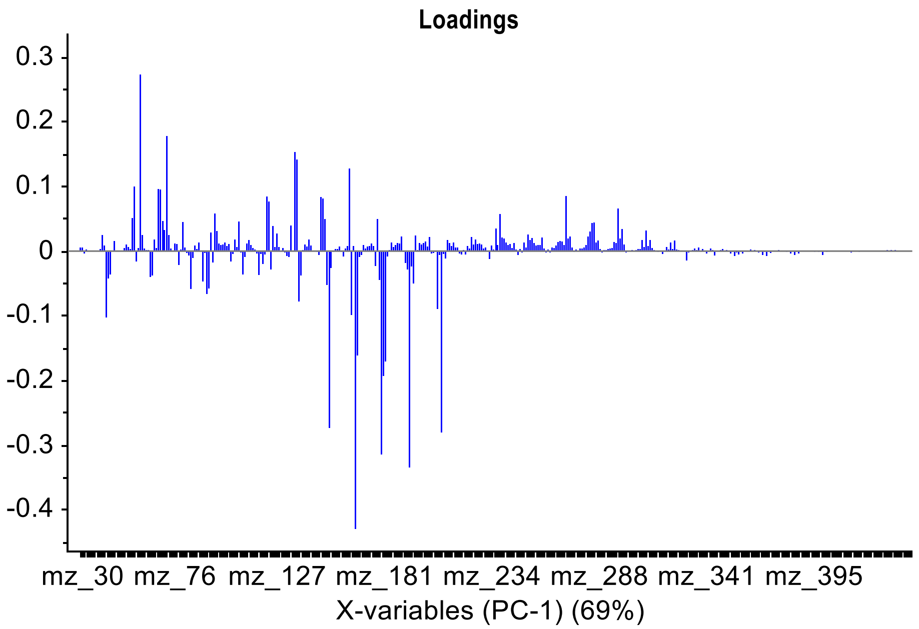
Loadings
0.3
0.2
0.1
0
-0.1
-0.2
-0.3
-0.4
mz_30 mz_76 mz_127 mz_181 mz_234 mz_288 mz_341 mz_395
X-variables (PC-1) (69%)

G)

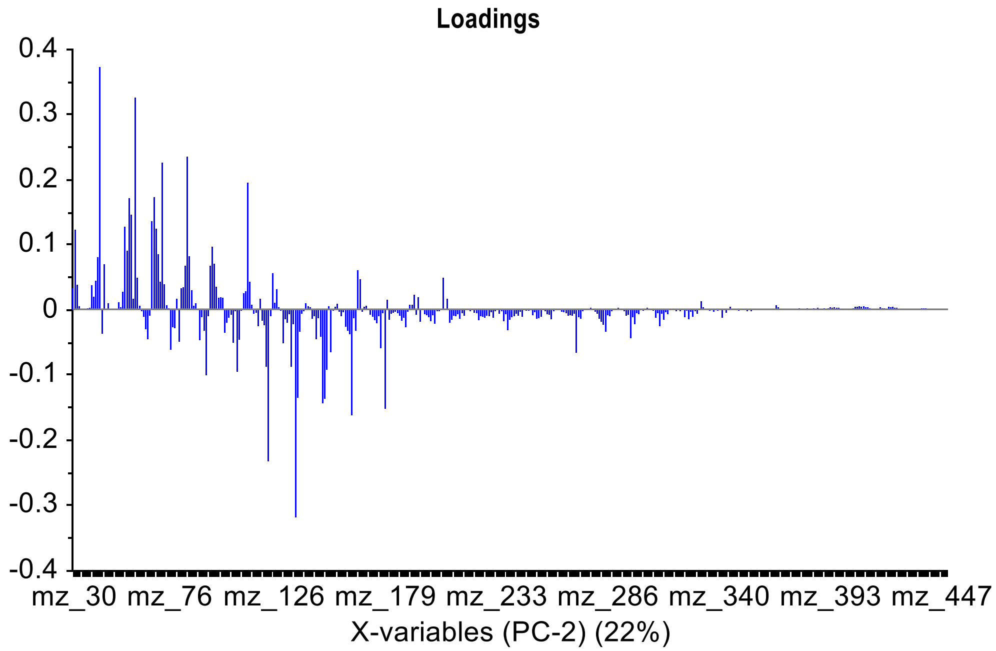
Loadings
0.4
0.3
0.2
0.1
0
-0.1
-0.2
-0.3
-0.4
mz_30 mz_76 mz_126 mz_179 mz_233 mz_286 mz_340 mz_393 mz_447
X-variables (PC-2) (22%)

Supplementary Figure 2. Aspen Control analysis throughout course of experiment. A) average spectrum B) variance of each ion.

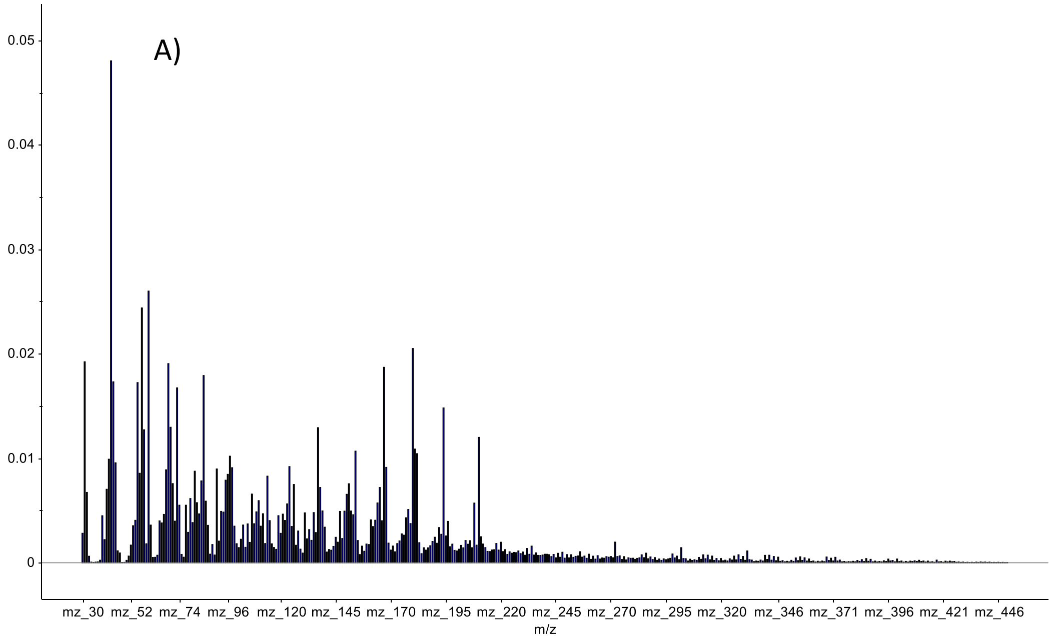

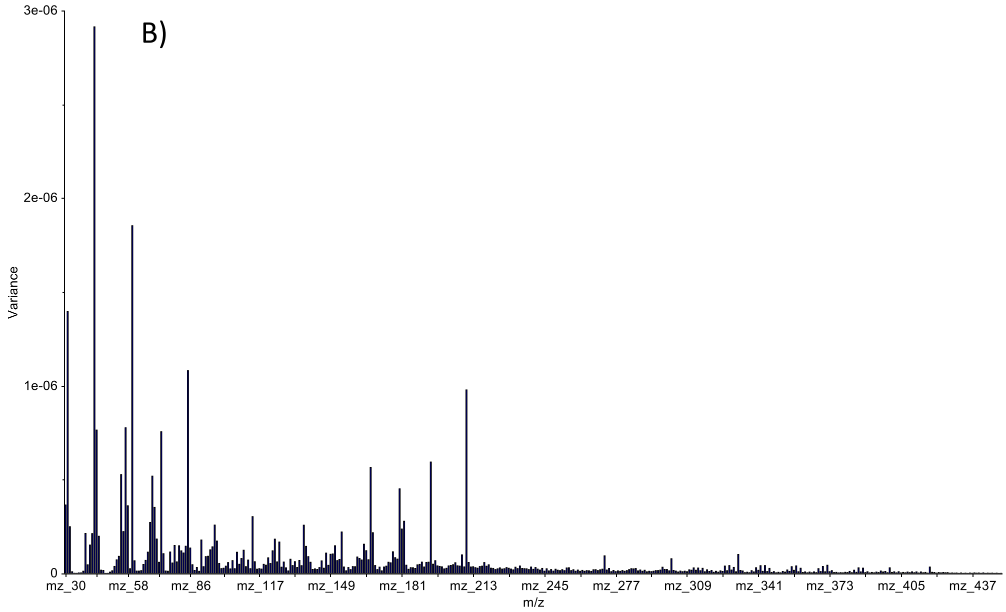

Supplementary Figure 3. PC-1 as a function of time using Control Aspen spectra (TIC-normalized) over the course of analysis by py-MBMS. X-axis values correspond to tray number (chronological sequence) in which the aspen sample was analyzed. >120 trays were analyzed over the course of 6 weeks.

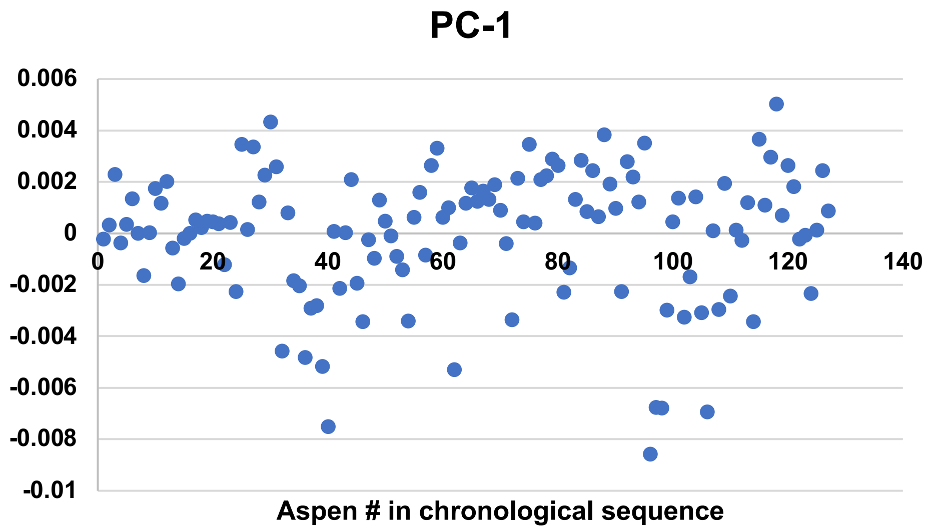

Supplementary Figure 4. PCA analyses of replicate 7x7 poplar sample analyses. Colors show replicates prior to tray and TPS correction.

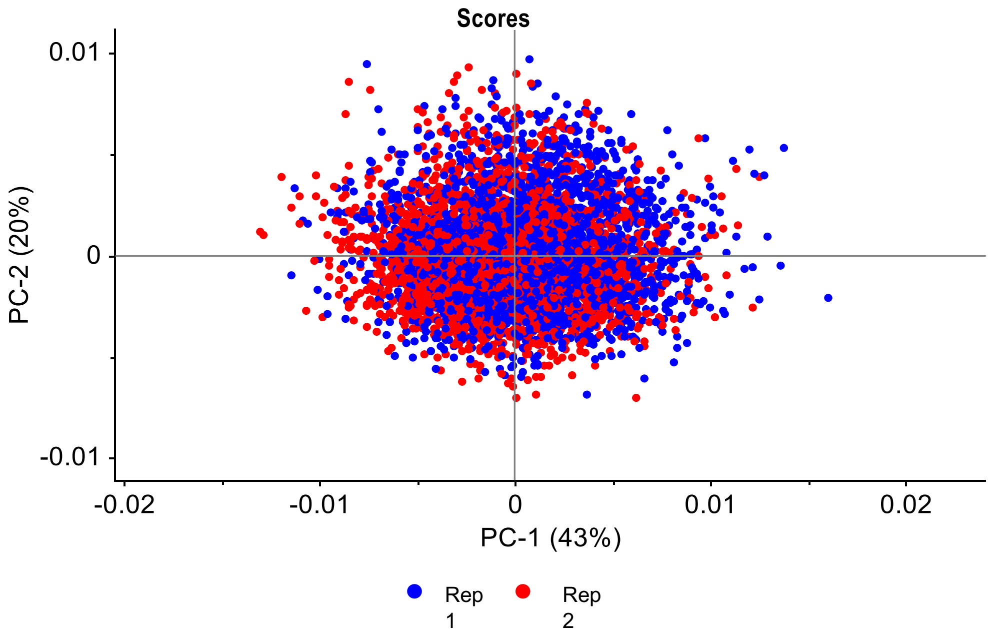

Supplementary Figure 5. TPS-predicted spectra hierarchical clustering dendrogram where colors show separation of 7 clusters corresponding to K-means clusters (Supplementary Figure 9).

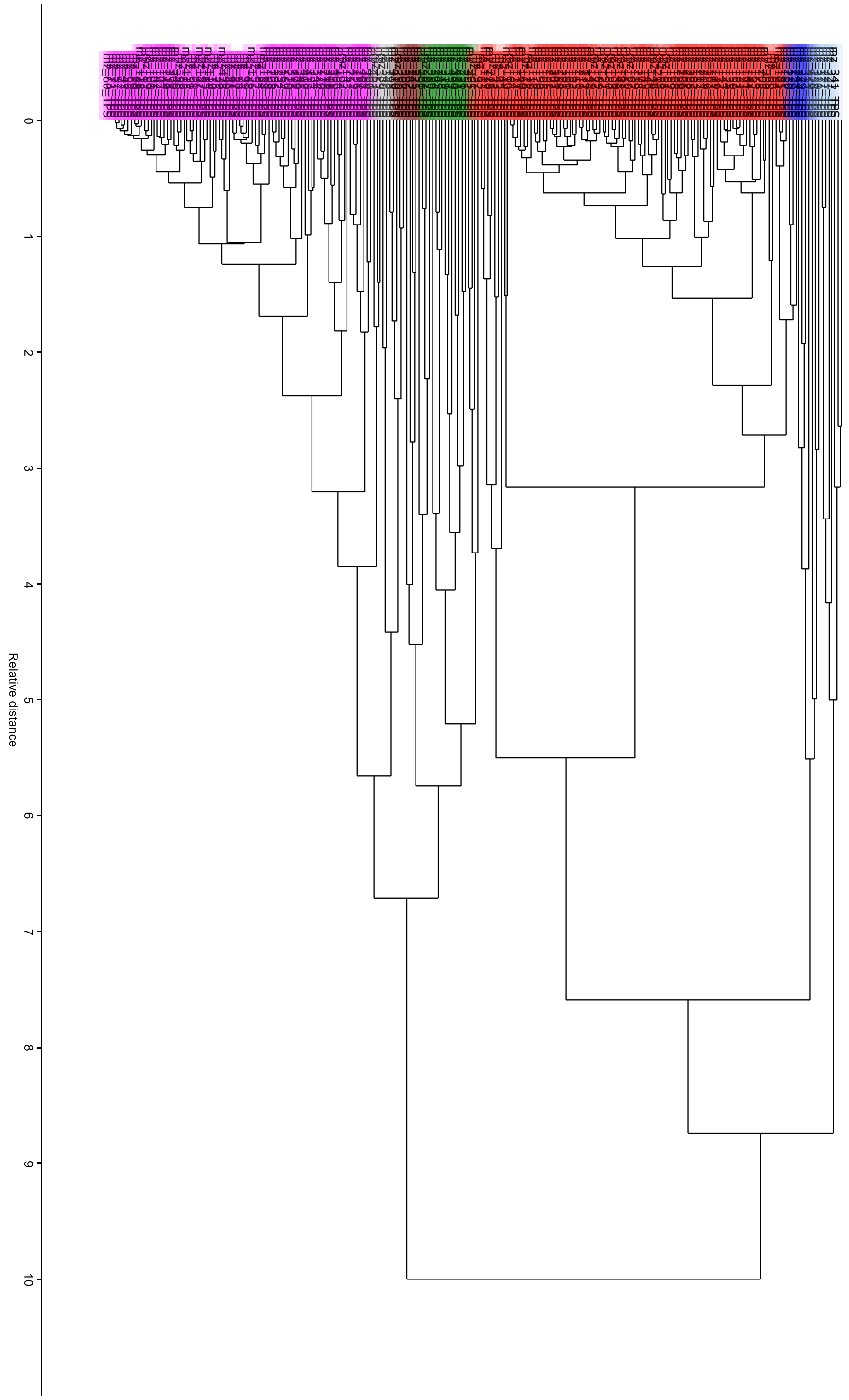

Supplementary Figure 6. PCA of entire population with spatial location of sample highlighted prior to TPS-tray correction of spectra and after TPS-tray corrections.

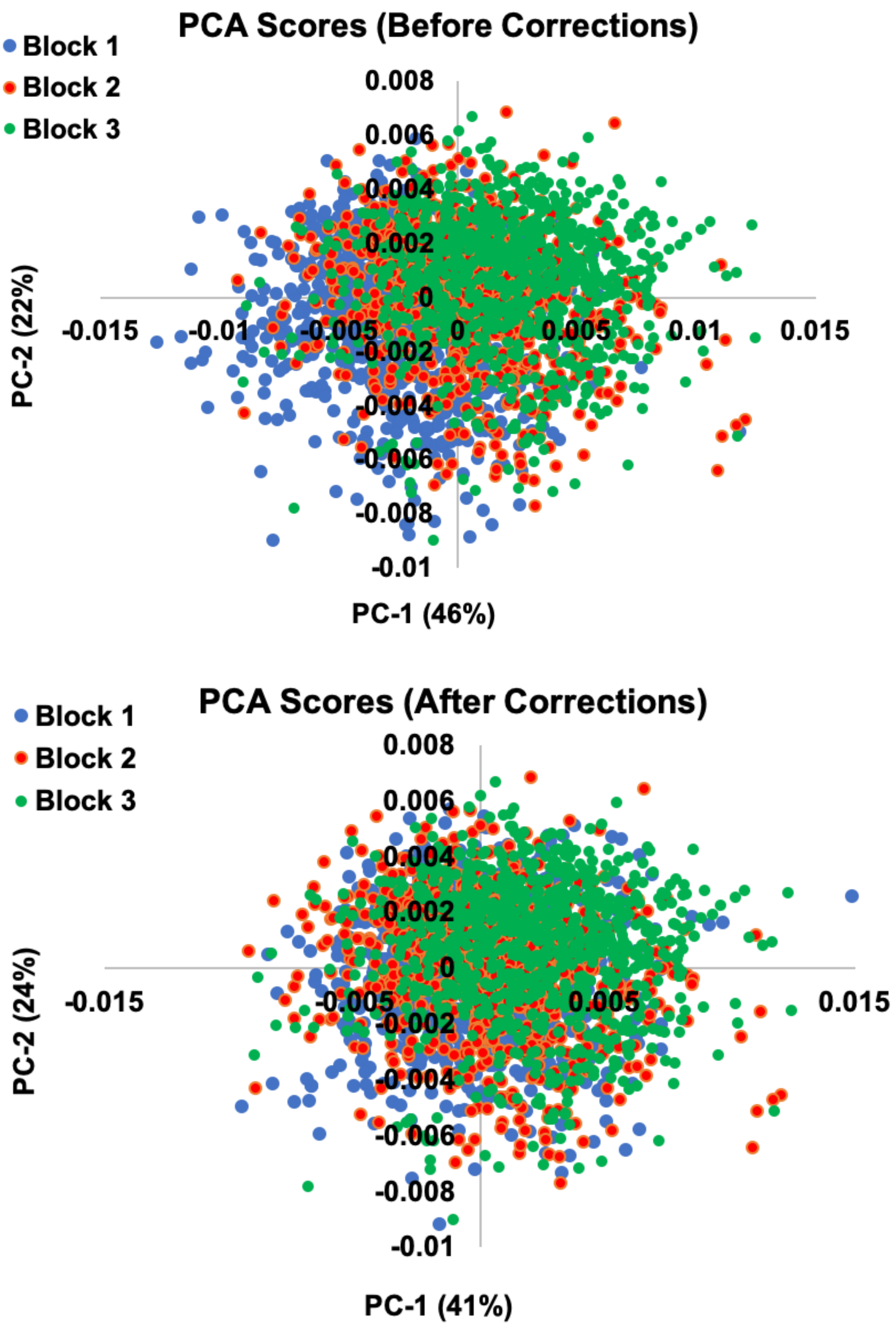

Supplementary Figure 7. Broad-sense heritability of ions in py-MBMS spectra with and without tray (A) and TPS (B) correction.

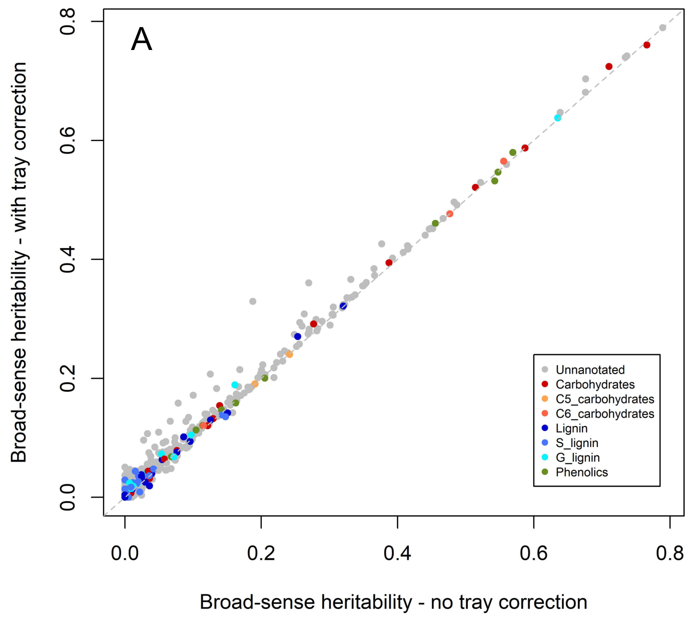

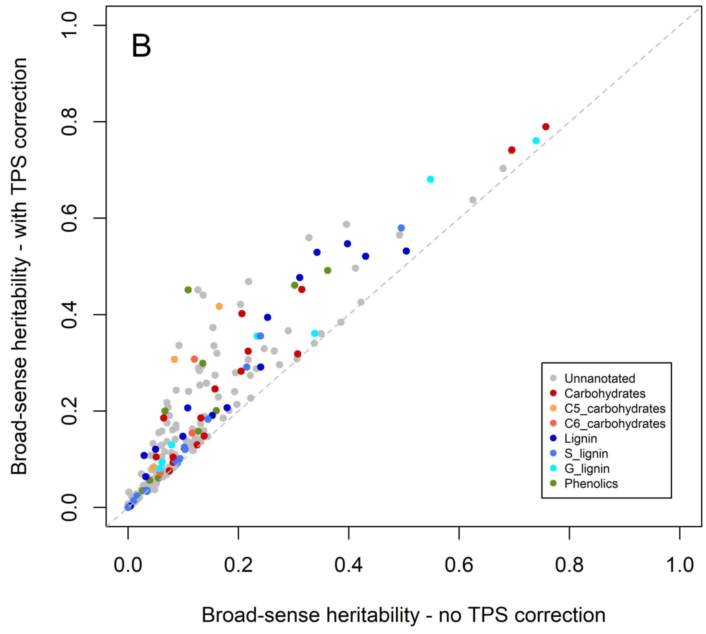

Supplementary Figure 8. Variance of ions in py-MBMS spectra explained from maternal and paternal effects.

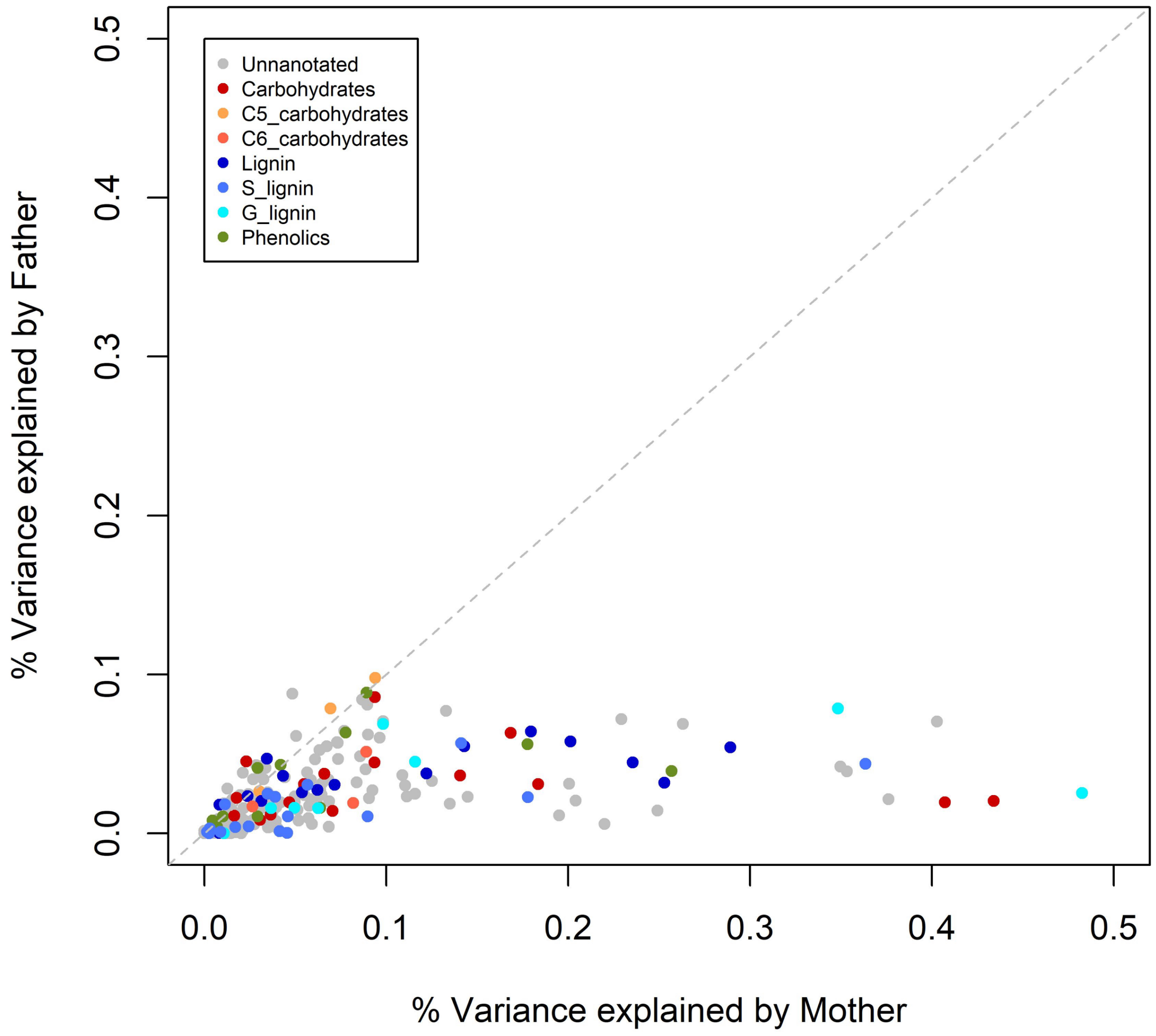

Supplementary Figure 9. K-means cluster analysis of ions in spectra from 7x7 pedigree.

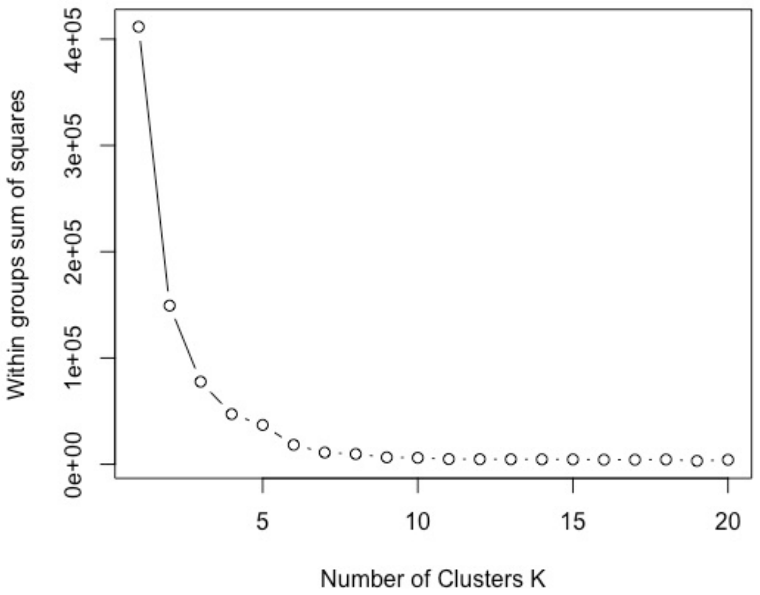

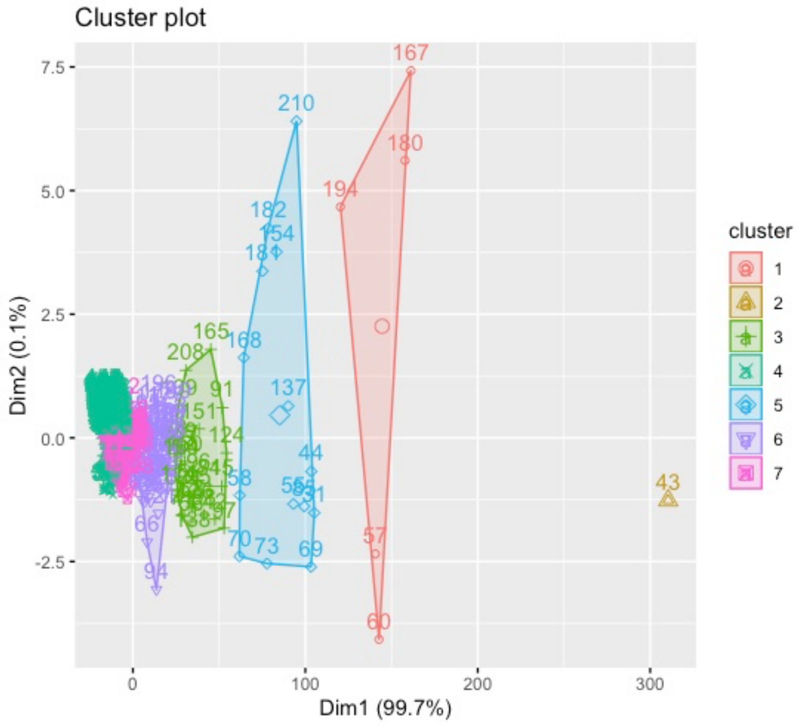

Supplementary Figure 10. Hierarchical Clustering (complete linkage, Spearman's rank correlation distance method) of ions from spectra of entire population after tray and TPS correction. EK# shown corresponding to different colors of ions in dendrogram corresponding with Supplementary Table 5.

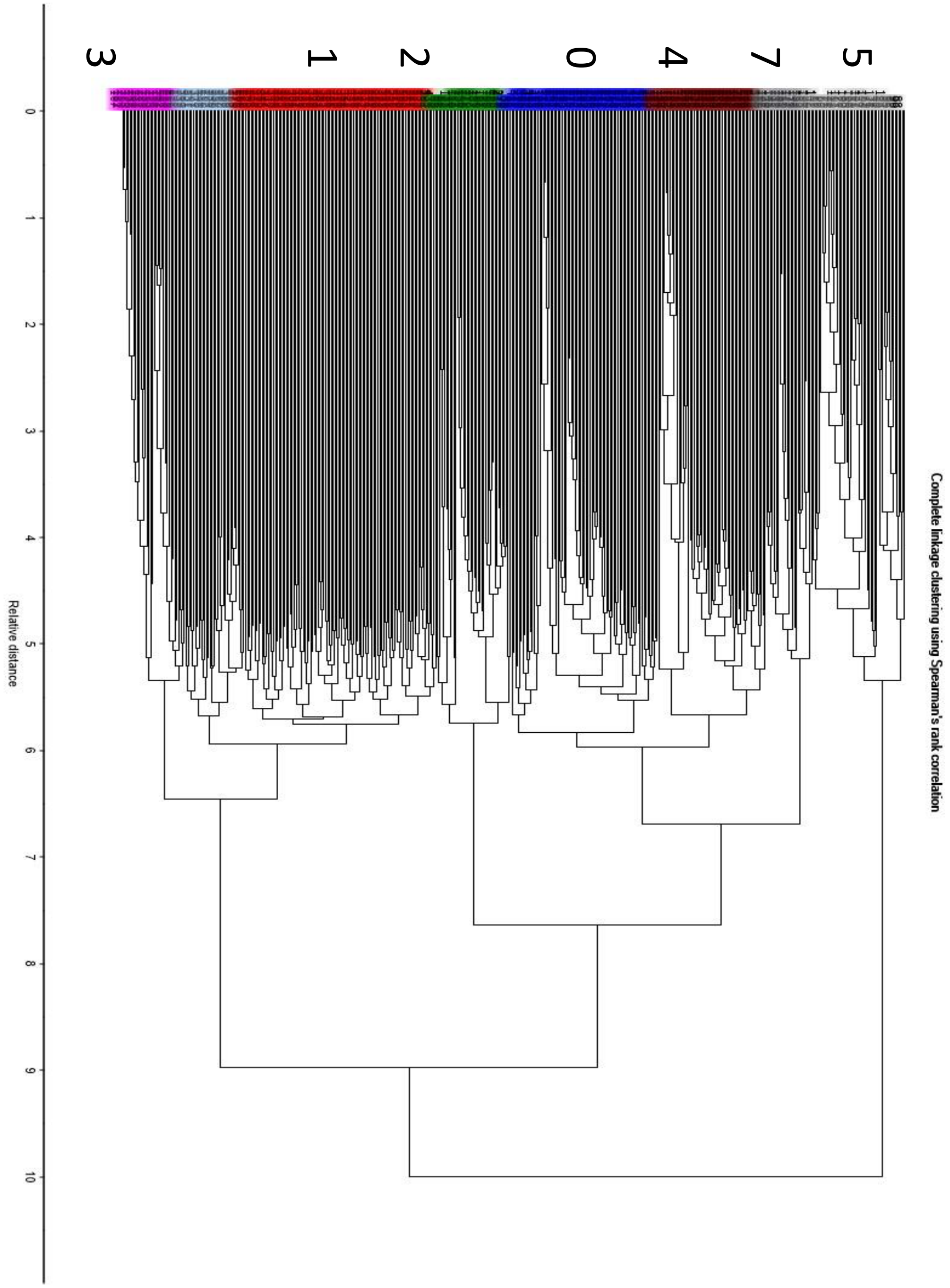

Supplementary Figure 11. Lignin content and composition differences based on maternal and paternal relationships.

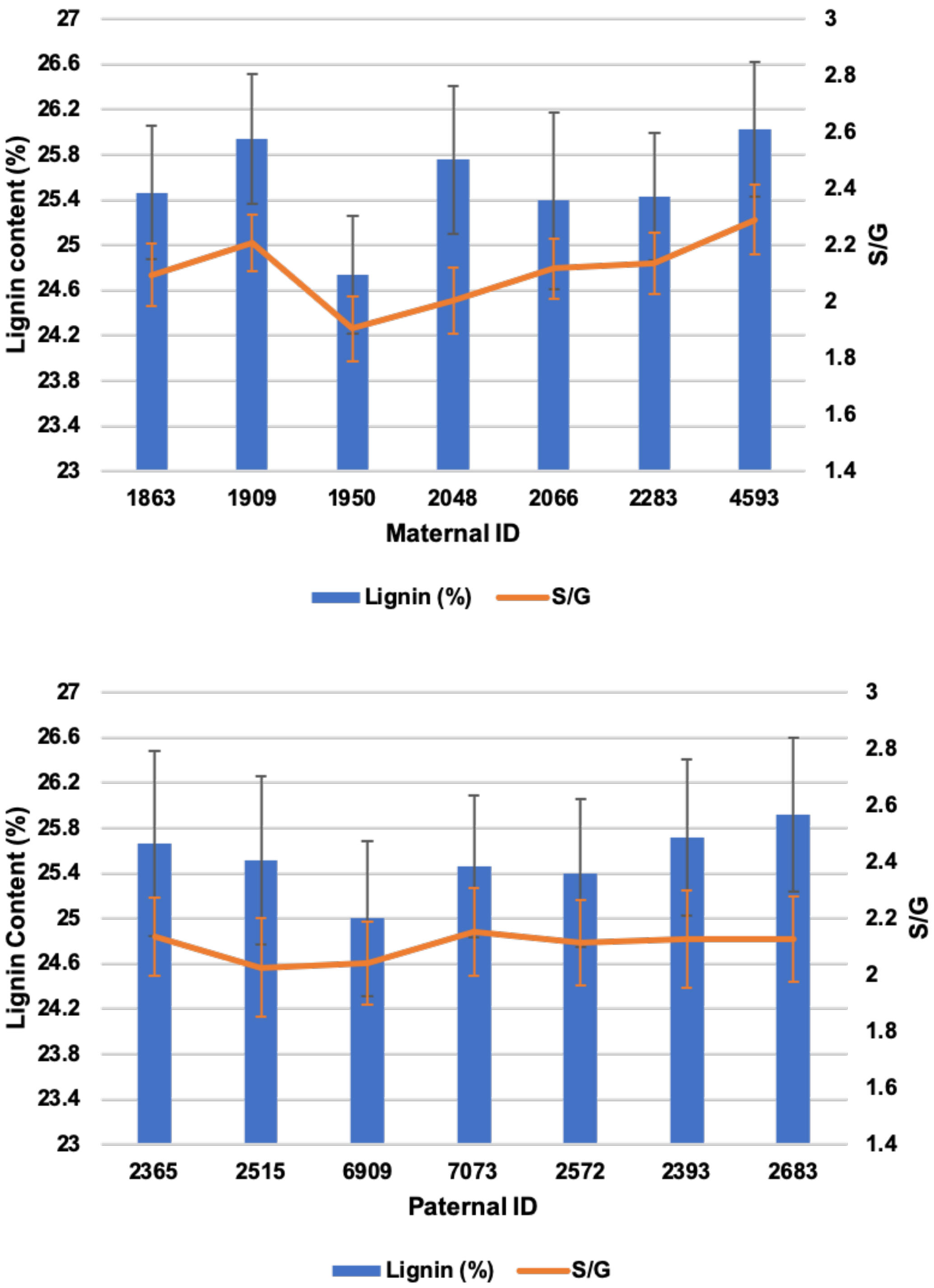

Supplementary Figure 12. Clusters of samples after tray and TPS correction (Ward’s Euclidean, 7 clusters).

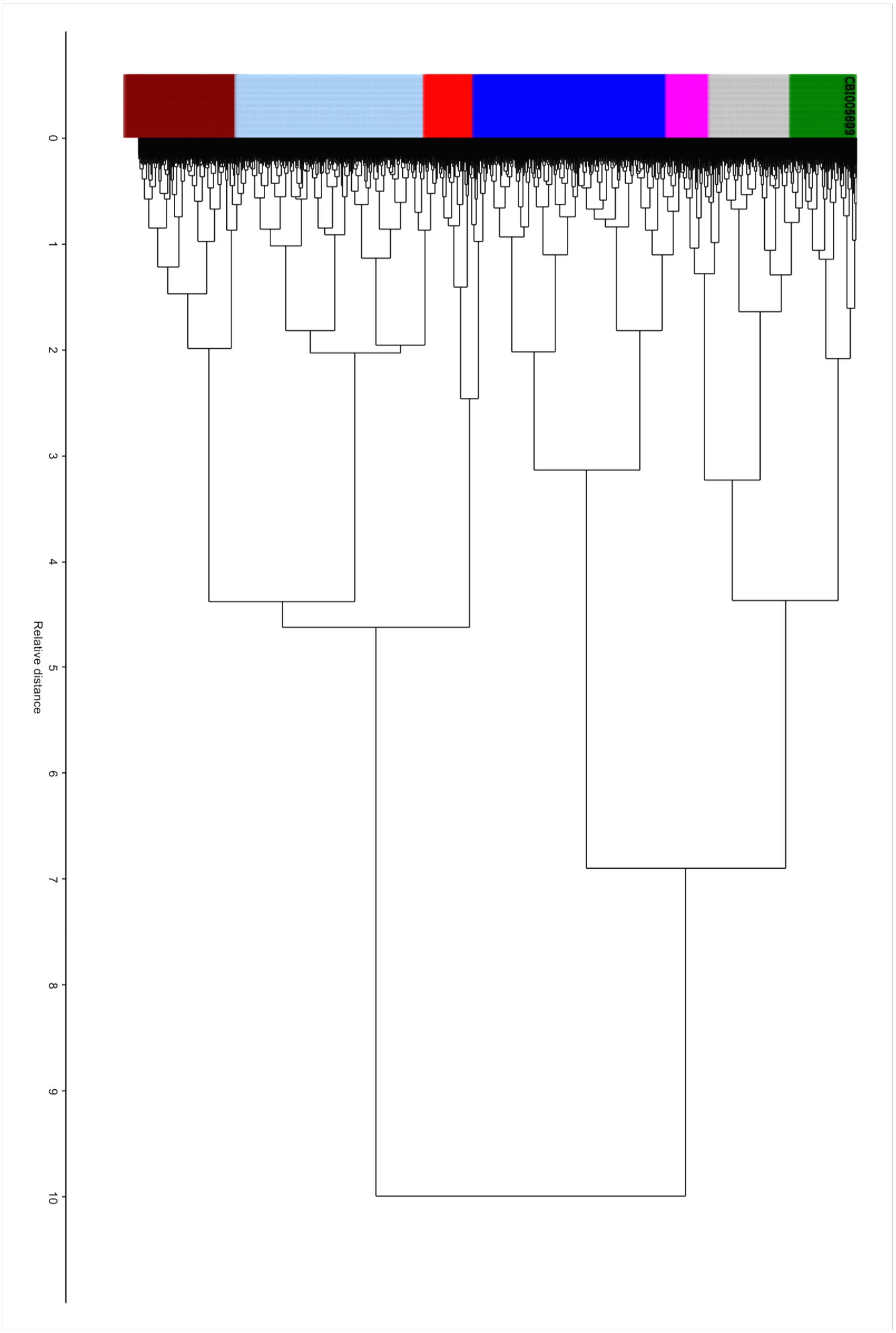

Supplementary Figure 13. K-means clustering of samples based on spectra after clonal averaging from 7x7 *P. trichocarpa* pedigree.

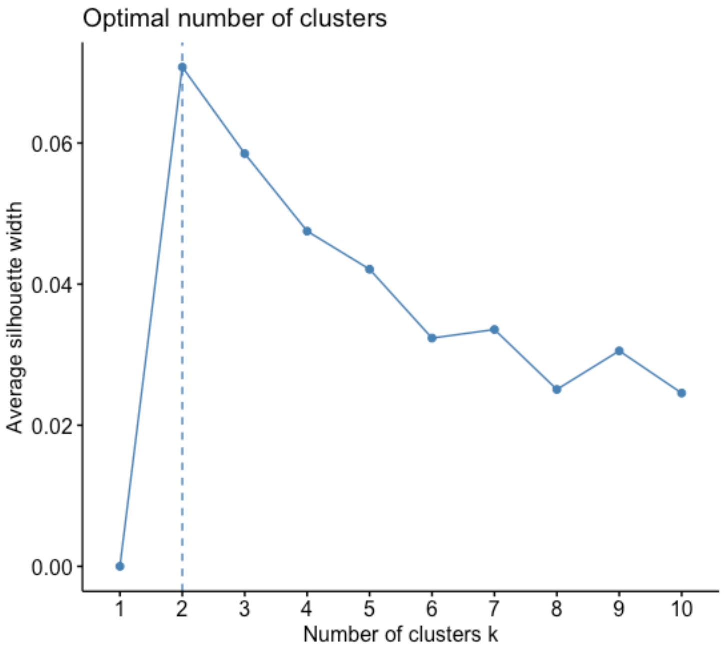

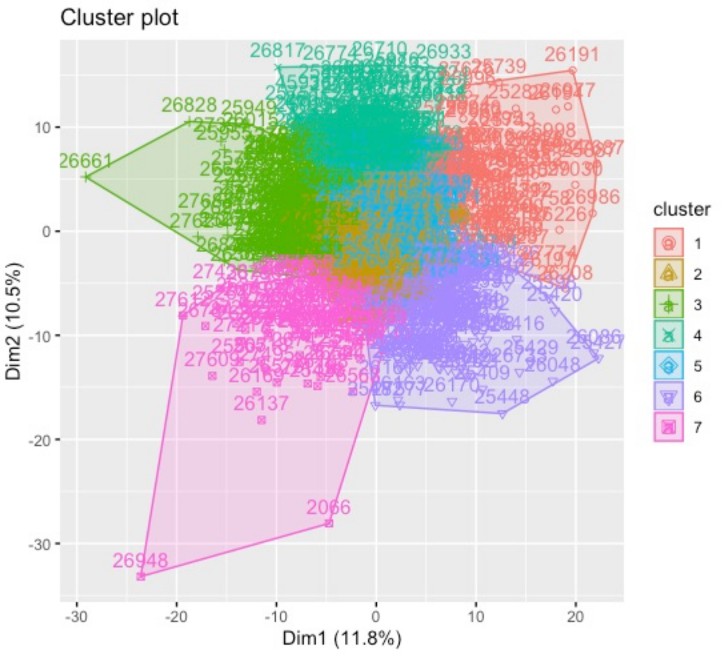

Supplementary Figure 14. PCA projection of corrected spectra from population after clonal averaging showing clustering associated with *m/z* 94 abundance (blue square being lowest, red circle is moderate and green triangle is highest intensity of *m/z* 94).

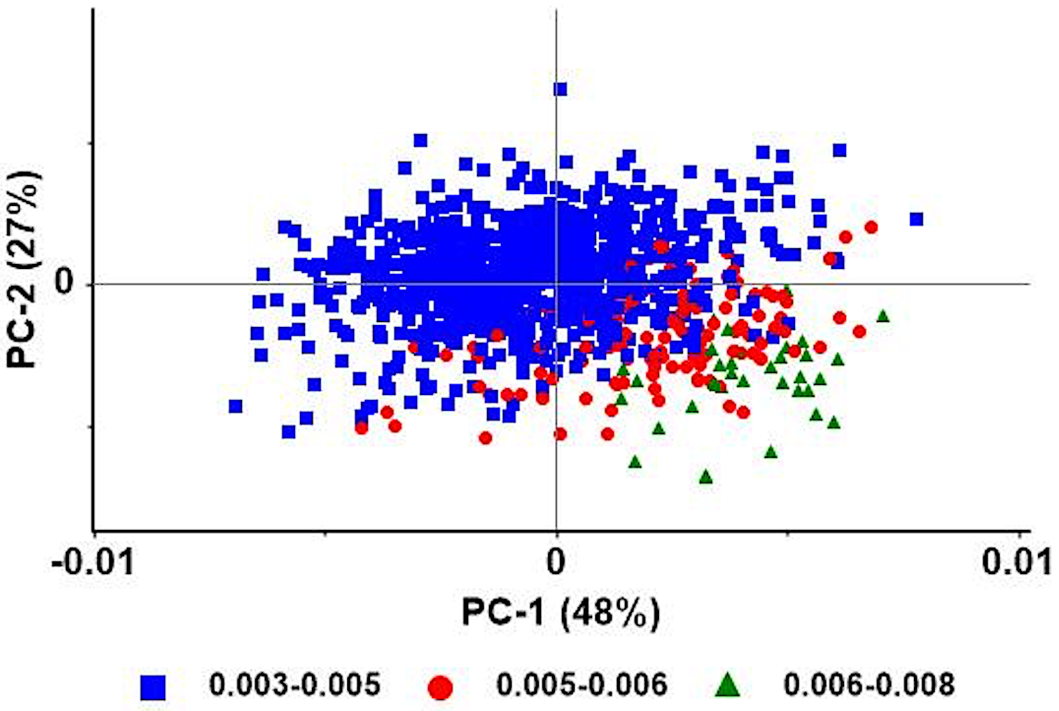
PC-2 (27%)
0
-0.01
0
0.01
PC-1 (48%)
0.003-0.005
0.005-0.006
0.006-0.008

Supplementary Figure 15. Comparison of phenolic (m/z 94) and S lignin (m/z 210) derived ions across half-sib families, error shows standard deviation within each family.

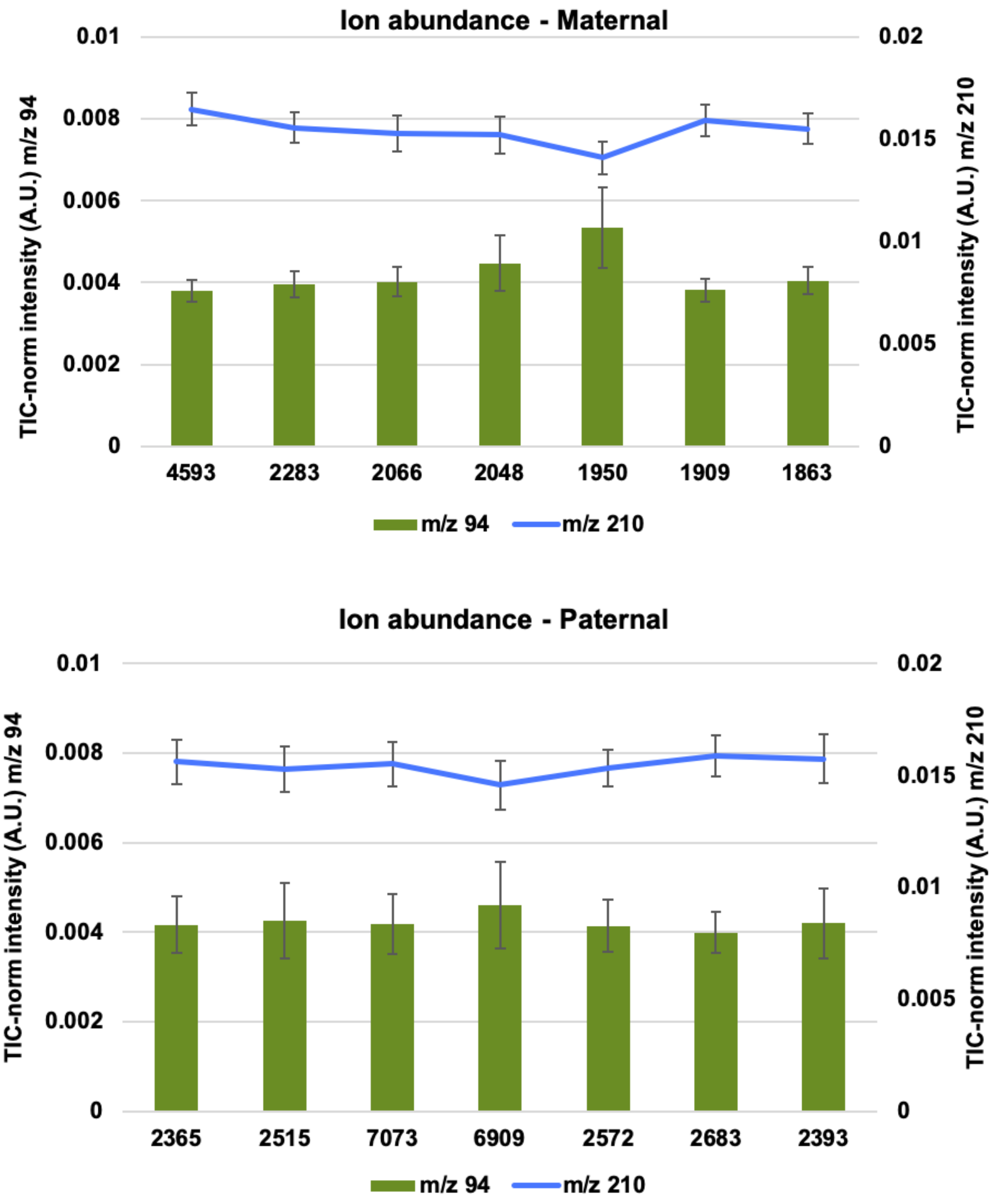

Supplementary Figure 16. Block diagram of the progeny field design.

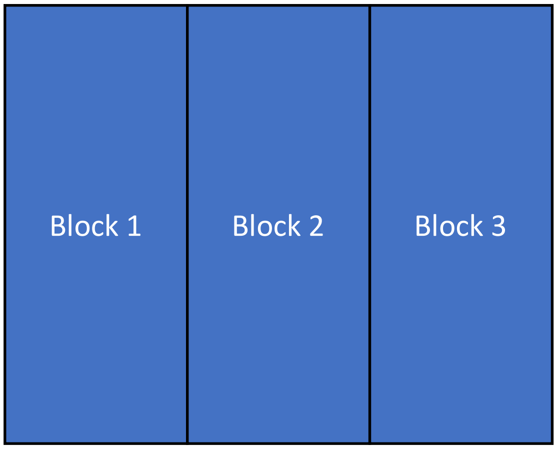

Supplement: Supplementary file 1 — Additional file 1: Table S1. Quality control metrics for validation and comparison of sample analysis before and after cleaning on the basis of Aspen controls. Parentheses indicate standard deviation. Table S2. Summary of quality control composition metrics determined for P. trichocarpa pedigree samples. Parentheses indicate standard deviation. Values are determined prior to TPS and tray correction. *Two samples were analyzed once. Table S3. Annotations of ions in py-MBMS spectra (supplied as separate file). Table S4. Summary of variance, maternal and paternal effects of each ion after TPS and tray correction (supplied as separate file). Table S5. Comprehensive list of ions in 8 clusters from HC-SRC (supplied as separate file). Table S6. Additional identifier information associated with parents of the P. trichocarpa population. Table S7. Estimates of broad sense heritability for studies in the Salicaceae that used py-MBMS to estimate total lignin and/or S/G ratios for species from the Salicaceae. Figure S1. PCA of standards analyzed throughout course of poplar analysis. Scores plots with numbers at each point indicate the tray (chronological sequence) number associated with particular sample. A) Entire scores plot of all standards, B) zoomed scores plot of region consisting primarily of Center for Bioenergy Innovation (CBI) poplar, Aspen and Poplar 068, C) scores of Loblolly Pine 6G1 standard, D) scores of NIST 8492 (Populus Deltoides), E) scores of Poplar 93968, F) corresponding loadings of spectra from standards only plotted in spectral format for PC-1 and G) loadings for PC-2 of the standard spectra only. “Pop” corresponds to poplar and “Lob” corresponds to Loblolly pine. Figure S2. Aspen Control analysis throughout course of experiment. A) average spectrum B) variance of each ion. Figure S3. PC-1 as a function of time using Control Aspen spectra (TICnormalized) over the course of analysis by py-MBMS. X-axis values correspond to tray number (chronological sequenc [file 13068_2021_1908_MOESM1_ESM.pdf]
